# Hulless barley polyphenol extract inhibits adipogenesis in 3T3-L1 cells and obesity related-enzymes

**DOI:** 10.3389/fnut.2022.933068

**Published:** 2022-08-04

**Authors:** Xianfeng Deng, Bi Chen, Qin Luo, Xingru Zao, Haizhe Liu, Yongqiang Li

**Affiliations:** ^1^College of Food Science and Technology, Yunnan Agricultural University, Kunming, China; ^2^School of Life and Health Science, Kaili University, Kaili, China

**Keywords:** hulless barley, polyphenols, enzymes, 3T3-L1 cells, adipogenesis

## Abstract

Obesity is characterized by excessive lipid accumulation, hypertrophy, and hyperplasia of adipose cells. Hulless barley (*Hordeum vulgare* L. var. nudum Hook. f.) is the principal crop grown in the Qinghai-Tibet plateau. Polyphenols, the major bioactive compound in hulless barley, possess antioxidant, anti-inflammatory, and antibacterial properties. However, the anti-obesity effect of hulless barley polyphenol (HBP) extract has not been explored. Therefore, the current study assessed the impact of HBP extract on preventing obesity. For this purpose, we evaluated the inhibitory effect of HBP extract against obesity-related enzymes. Moreover, we investigated the effect of HBP extract on adipocyte differentiation and adipogenesis through 3T3-L1 adipocytes. Our results demonstrated that HBP extract could inhibit α-amylase, α-glucosidase (α-GLU), and lipase in a dose-dependent manner. In addition, HBP extract inhibited the differentiation of 3T3-L1 preadipocytes by arresting the cell cycle at the G0/G1 phase. Furthermore, the extract suppressed the expression of adipogenic transcription factors such as peroxisome proliferator-activated receptor γ (PPARγ), CCAAT/enhancer-binding protein α (C/EBPα), regulating fatty acid synthase (FAS), fatty acid-binding protein 4 (FABP4), and adipose triglyceride lipase (ATGL). It was also observed that HBP extract alleviated intracellular lipid accumulation by attenuating oxidative stress. These findings specify that HBP extract could inhibit obesity-related enzymes, adipocyte differentiation, and adipogenesis. Therefore, it is potentially beneficial in preventing obesity.

## Introduction

Obesity has become one of the most severe global health problems (1), associated with numerous metabolic diseases, such as type 2 diabetes (T2D), cardiovascular disease, fatty liver, insulin resistance, and several cancers (2). Obesity is an excessive or abnormal lipid accumulation characterized by increased adipose tissue due to an imbalance between energy intake and expenditure (3). Adipose tissue expansion is due to an increase in the number of adipocytes (hyperplasia) and cell size (hypertrophy). Specifically, hypertrophy is associated with adipogenesis, a process of differentiating preadipocytes into adipocytes and subsequent lipid accumulation (2). Therefore, adipogenesis plays a pivotal role in regulating overall fat mass. Murine 3T3-L1 preadipocytes are the well-characterized models for the assessment of preadipocyte differentiation, adipogenesis, and lipid accumulation (4).

Moreover, adipogenesis is a complex process regulated by numerous transcription factors, including CCAAT/enhancer-binding protein α (C/EBPα), peroxisome proliferator-activated receptor γ (PPARγ), fatty acid synthase (FAS), fatty acid-binding protein 4 (FABP4), and adipose triglyceride lipase (ATGL) during adipogenesis (5). During the preadipocyte differentiation process, PPARγ and C/EBPα control gene expression through cross-regulation. Furthermore, PPARγ and C/EBPα co-activate the expression of downstream target genes such as FAS, FABP4, and ATGL. Once they are expressed, fat droplets are aggregated and differentiated into adipocytes (6).

Increasing evidence has demonstrated that reducing food intake and improving physical activity can affect weight loss, but it is challenging for most individuals. In this sense, nutrition intervention can be a practical approach to obesity. Some authors have demonstrated the potential of natural phytochemicals, like phenolic compounds (3, 7) and β-carotenoids (8), as promising anti-obesity drug-like candidates. Phenolic compounds are important secondary metabolites (9), widely distributed among cereals (10), vegetables (11), and fruits (12). Furthermore, polyphenols can be divided into phenolic acids, flavonoids, lignans, and stilbenes based on their structure. Some authors have reported a variety of bioactivities and beneficial health effects of phenolic compounds, widely evaluated *in vitro* and *in vivo* models, including antioxidant, anti-obesity, anti-carcinogenic, and anti-inflammatory properties (13). Recent studies have demonstrated that phenolic compounds derived from fruits, vegetables, and grains can inhibit lipid accumulation during adipocyte differentiation and reduce the obesity caused by excessive lipid storage (3). Thus, phenolic compounds, an integral part of the human diet, have emerged as crucial phytochemicals in preventing and treating obesity (14).

At present, numerous traditional therapies are in practice against obesity, including inhibiting the enzymes responsible for carbohydrate and fat hydrolyses, such as α-amylase, α-GLU, and lipase (15). Many digestive enzyme inhibitors like acarbose for carbohydrates and orlistat for lipids have treated obesity by limiting energy intake (16). However, these chemically synthesized enzyme inhibitors have serious side effects. For example, acarbose causes abdominal pain, distension, and diarrhea, while the use of orlistat leads to body aches, headache, and nausea (17, 18). Therefore, it is necessary to discover naturally occurring digestive enzyme inhibitors with fewer secondary effects to prevent or reduce obesity. Several studies have shown that natural phenolic compounds could inactivate digestive enzymes such as α-amylase, α-GUL, and lipase through non-specific enzyme binding (19, 20). Therefore, natural polyphenolic compounds can manage obesity by inhibiting digestive enzyme activity. Cellular oxidative stress is an imbalance between oxidation and antioxidation. Excessive reactive oxygen species (ROS) production can induce oxidative stress and cell damage (21). Therefore, attenuating oxidative stress in adipocytes plays a critical role in preventing obesity.

Hulless barley (*Hordeum vulgare* L. var. nudum Hook. f.), also known as the naked barley, is the principal crop cultivated in the Qinghai-Tibet Plateau. It is also the staple food of local farmers and herdsmen (22). The Qinghai-Tibet Plateau has a high altitude, hypoxia, low temperatures, and intense ultraviolet rays, promoting the synthesis and accumulation of phenolic compounds (23). Some studies have investigated the composition and the antioxidant activity of phenolic compounds in hulless barley. In a previous report, the hulless barley contained more protein, starch, and β-glucan, as well as higher total phenolic content and antioxidant activity compared to regular hulled barley (24). In addition, the antioxidant activity of free phenolic extracts from hulless black barley was improved by lactic acid bacteria fermentation (25). Moreover, Qingke β-glucan not only ameliorated high fat-diet-induced obesity but also attenuated capsaicin-induced gastrointestinal injury in Kunming mice (26). However, the possible role of phenolic compounds from hulless barley against obesity has not been elucidated. Therefore, the objectives of this study include: (1) the assessment of the antioxidant ability of the hulless barley polyphenol (HBP) extract and the corresponding inhibitory effects against three digestive enzymes. (2) identification of the HBP extract on lipid accumulation in 3T3-L1 adipocytes. (3) evaluation of the effect of HBP extract on the expression of genes and proteins associated with lipid metabolism.

## Materials and methods

### Chemicals and reagents

The chemicals and reagents for the study included Fetal bovine serum (FBS), Dulbecco's Modified Eagle Medium (DMEM), Dimethyl sulfoxide (DMSO), penicillin-streptomycin, insulin, dexamethasone, 3,5-dinitrosalicylic acid (DNS), 3-isobutyl-1-methylxanthine (IBMX), p-nitrophenyl phosphate (pNPP), 3-(4,5-dimethylthiazol-2-yl)-2,5-diphenyltetrazolium bromide (MTT), p-nitrophenyl glucopyranoside (pNPG), Oil red O, Formaldehyde phosphate, Absolute ethanol, Sodium cholate, Gum Arabic, Methanol, Formic acid, Folin–Ciocalteu (Merck, Darmstadt, Germany), Na_2_CO_3_, α-Amylase, α-GLU, Lipase, PPARγ, C/EBPα, FAS, FABP4, and ATGL, and were purchased from the Nanjing Jiancheng Bioengineering Institute (Nanjing, China).

### Preparation of HBP extract

Hulless barley (“Changhei” hulless barley) was obtained from the Institute of Yunnan Diqing Tibetan Autonomous Prefecture Agricultural Sciences (Yunnan, China). First, HBP extract was prepared using the previously reported method of Zhu et al. with certain modifications (27). In brief, 1g of hulless barley powder was added to 10 mL of the extraction solvent, 80% methanol containing 0.1% formic acid (*V*/*V*). Next, the mixture was sonicated in an ice water bath for 30 min. This procedure was repeated twice using the same solvent. Finally, the three obtained supernatants were combined after centrifugation (Model TGL-20M, Hunan, China) at 8,000 r/min for 15 min. Subsequently, the supernatant was fixed at 50 mL volume with extraction solvent and stored away from light at −20°C for later use. This extract solution was used to determine the total phenolic content (TPC) and the inhibition of α-amylase, α-GUL, and lipase.

The quantification of total phenolics was estimated using Folin-Ciocalteu colorimetric method as previously reported by Li et al. (28). The TPC was calculated by using a standard curve made with ferulic acid and expressed as micrograms (μg) of ferulic acid equivalent (FAE) per gram of dry weight (DW) (μg FAE/mL DW). Detailed information on the TPC estimated during the present study is available in Supplementary Table 2.

### Enzyme inhibition assays

#### α-amylase inhibition

The inhibition of α-amylase was evaluated using a previous method with minor changes (29). In brief, 500 μL of HBP extract was mixed with 500 μL of porcine pancreatic α-amylase (13 U/mL) at different concentrations (12.5–1,000 μg FAE/mL) using a previously prepared phosphate buffer (0.1 mol/L, pH 6.9), and incubated for 10 min at 37°C. Then, 500 μL of gelatinized starch solution (1%, *m*/*V*) was added to the mixture and incubated for another 10 min at 37°C. Subsequently, 1 mL of 3,5-dinitrosalicylic acid (DNS) reagent was added to the mixture and incubated for 5 min in a boiling water bath. Finally, the absorbance was measured at 540 nm. The inhibitory activity of α-amylase was calculated using Equation (1).


(1) $$Inhibition(\%)=\left(1-\frac{A_{s}-{A^{\prime }}_{s}}{A_{c}-{A^{\prime }}_{c}}\right)\times 100$$

Where *A*_*s*_ is the absorbance of the sample, enzyme, substrate, and DNS; *A*${}_{s}^{,}$ is the absorbance of the sample, substrate, and DNS; *A*_*c*_ is the absorbance of the buffer solution, enzyme, substrate, and DNS; *A*${}_{c}^{{}^{\prime }}$ is the absorbance of the buffer, substrate, and DNS.

#### α-GLU inhibition

Using a previous method with some modifications (30, 31), the α-GLU inhibitory activity was measured in a 96-microplate reader. In brief, 50 μL of HBP extract at different concentrations (12.5–1,000 μg FAE/mL) and 50 μL α-GLU (1U/mL) prepared using phosphate buffer (0.1 mol/L, pH 6.9) were added to a 10-mL tube. After incubation for 10 min at 37°C, 50 μL of 5 mmol/L pNPG (dissolved in phosphate buffer 0.1 mol/L, pH 6.9) was included for initiating the reaction and incubated for 10 min at 37°C. Then 2 mL of 0.1 mol/L Na_2_CO_3_ solutions was added to stop the reaction. The absorbance was measured at 405 nm, and the inhibition rate of α-GLU was calculated using Equation (2). In both the assays, acarbose (1 mg/mL, dissolved with distilled water) was the positive control for α-amylase and α-GLU.


(2) $$Inhibition(\%)=\left(1-\frac{A_{s}-{A^{\prime }}_{s}}{A_{c}-{A^{\prime }}_{c}}\right)\times 100$$

Where, *A*_*s*_ is the absorbance of the sample, enzyme, pNPG, and Na_2_CO_3_; *A*${}_{s}^{,}$ is the absorbance of the sample, pNPG, and Na_2_CO_3_; *A*_*c*_ is the absorbance of the buffer solution, enzyme, substrate, pNPG, and Na_2_CO_3_; *A*${}_{c}^{{}^{\prime }}$ is the absorbance of the buffer, pNPG, and DNS.

#### Lipase inhibition

The method of measuring the inhibition of pancreatic lipase was based on slight modifications of a previous procedure (19, 32). First, 40 μL of 0.1 M phosphate buffer (pH 6.9) was added into a 10-mL tube. Then, 40 μL of HBP extract at 12.5 to 1,000 μg FAE/mL concentrations was mixed with 40 μL of the pancreatic lipase solution (2.5 mg/mL) dissolved in phosphate buffer, and the mixture was incubated for 15 min at 37°C. After incubation, 20 μL of pNPP (1 mol/L) mixed with isopropanol was included in each tube and incubated for another 15 min at 37°C. After incubation, 100 μL of an anhydrous ethanol solution was added to stop the reaction. Finally, the absorbance was recorded at 405 nm, and orlistat was included as a positive inhibitor. The inhibition rate of lipase was calculated using Equation (3).


(3) $$Inhibition(\%)=\left(1-\frac{A_{LSOPC}-A_{blank}}{A_{test}-A_{control}}\right)\times 100$$

where *A*_LSOPC_ is the absorbance of the sample, enzyme, and Pnpp; *A*_blank_ is the absorbance of the sample and pNPP; *A*_test_ is the absorbance of the buffer, enzyme, and pNPP; *A*_control_ is the absorbance of the buffer and pNPP.

IC_50_ (the half-maximal inhibitory concentration) is defined as the concentration of the extract required to inhibit 50% of the enzyme activity (33). The IC_50_ values of the α-amylase, α-GLU, and lipase were determined using the Graph Pad Prism software version (8.0.2).

### Cell culture and viability assays

#### Cell culture and differentiation induction

3T3-L1 murine preadipocytes were obtained from the ATCC (Manassas, VA, USA). The cell culture was performed according to the method described by Hyun et al. (34), with certain modifications. The complete cell medium was developed by adding 10% fetal bovine serum (HyClone) and 1% penicillin-streptomycin (Fisher) to DMEM for the 3T3-L1 preadipocyte culture and adipocyte differentiation. Subsequently, the cells were seeded in 50 mL culture flasks. Then, 3 mL of pre-warmed complete culture solution (37°C, 5 min) was added to the flasks. Finally, the cell culture flasks were incubated at 37°C in a 5% CO_2_ incubator for 2 to 3 days. Differentiation experiments were undergone when the cell density reached 90%.

The differentiation medium (MDI) containing 10 mmol/L dexamethasone (DEX), 0.5 mM IBMX, and 0.1 mg/mL insulin induced the preadipocyte differentiation. After incubating for 48 h, the MDI was replaced with a differentiation medium containing 0.1 mg/mL insulin and cultured for another 48 h. After that (day 4), the culture medium was discarded, the cells were cultured in the complete medium until day 8, and the medium was changed every 48 h. Furthermore, the sample groups were treated with a differentiation medium containing variable concentrations (5, 10, and 25 μg FAE/mL) of the HBP extract. The differentiation medium supplemented with N-acetylcysteine (NAC) was a positive control.

#### Cytotoxicity

The cytotoxicity of 3T3-L1 preadipocytes and mature adipocytes was determined using the MTT colorimetric assay. Cytotoxicity assay was performed based on the procedure by Liu et al. (35) with a few modifications. First, 3T3-L1 preadipocytes were seeded into 96-well plates at a density of 2 × 10^4^ cells/mL with various concentrations (0–100 μg FAE/mL) of HBP extract for 24 h. Afterward, the wells were washed using PBS three times, and MTT at 5 mg/mL concentration was added. After 4 h of incubation, the liquid medium was discarded and 100 μL of DMSO was added. The absorbance was measured at 490 nm with a microplate spectrophotometer. 3T3-L1 preadipocytes were differentiated using MDI and HBP extract for the mature adipocytes. After incubation (day 8), cells were collected and determined using the MTT colorimetric assay described above.

### Adipocyte differentiation assay

#### Oil red O staining and lipid quantification

Oil Red O staining in 3T3-L1 cells was performed based on a previously reported method with minor modifications (36). For mature adipocytes, the solution was fixed with 10% formaldehyde phosphate solution at room temperature for 1 h and washed with phosphate buffer solution two times. Next, the fixed cells were stained using 0.5% Oil red O solution for 1 h. Subsequently, the dye retained in adipocytes was extracted using 100% isopropanol, and the absorbance was measured at 510 nm.

#### Triglycerides (TG) quantification

Mature adipocytes were extracted and lysed in lysis buffer (0.5% Triton X-100 in PBS) for 30 min on an icebox. Then, the combined cell lysate was incubated for 10 min at 37°C. TG content was measured with a triacylglycerol enzymatic kit (Nanjing Jiancheng Bioengineering Institute, China) and normalized with the protein concentration of cell lysates. The protein concentration of the lysates was determined using a BCA protein assay kit (Beyotime, Haimen, Jiangsu, China).

### Flow cytometry

3T3-L1 preadipocytes were treated with MDI in the presence or absence (control) of HBP extract (5, 10, and 25 μg FAE/mL) for 24 h. Subsequently, cells were fixed in 70% ethanol for 12 h at 4°C. After 12 h, the cells were washed with cold PBS, mixed with 0.2 mg/mL propidium iodide (PI) containing 0.5% Triton X and 0.5 mg/mL R Nase, and incubated for 30 min at 37°C (protected from light). Flow cytometry was performed after staining with 500 μL PI using a flow cytometer (Accuri C6 Plus Cytometer, USA).

### Determination of cellular antioxidant activity

The intracellular reactive oxygen species (ROS) was determined by a procedure described previously (37). The 3T3-L1 preadipocytes were seeded into 96-well plates at a density of 2 × 10^4^ cells/well. After culturing the mature adipocytes using the method described above, the cells were washed two times with PBS. First, 100 μL of PBS solution containing 10 μmol/L 2,7-dichlorofluorescein-diacetate (DCFH-DA) was added to each well in a 5% CO2 concentration incubator for 30 min at 37°C. After that, 100 μL PBS was added to the 96-well plates. The fluorescence intensity was measured with a multimode detector using an excitation wavelength of 488 nm and an emission wavelength of 525 nm. After induction for 8 days, the treated and the untreated mature adipocytes were washed. The contents of superoxide dismutase (SOD), catalase (CAT), glutathione (GSH), and malondialdehyde (MDA) were determined using the detection kit (Nanjing Jiancheng Bioengineering Institute, China) to identify the potential biomarker of oxidative stress.

### RNA Isolation, cDNA synthesis, and RT-qPCR

The extracted RNA was quantified using real-time quantitative PCR (RT-qPCR). Total RNA was extracted using the RNA extraction kit (Dongsheng Biotech, Guangzhou, China) based on the manufacturer's instructions. The cDNA was synthesized with 1 μg of the total RNA. The specific primers for RT-qPCR included PPARγ, C/EBPα, FAS, FABP4, and ATGL. The forward and reverse expression sequences of five particular primers are shown in Table 1. The qPCR amplification program was as follows: pre-incubation at 95°C for 10 min; amplification at 95°C for 10 s, annealing at 60°C for 20 s, and the final extension step at 72°C for 15 s. The transcription product levels were normalized using β-actin as the internal reference, and relative gene expression was calculated based on the 2^−Δ*ΔCt*^ method.

**Table 1:** Primer sequences used for RT-qPCR analysis.

**Gene**	**Forward primers**	**Reverse primers**
**PPARγ**	CTGTGAGACCAACAGCCTGA	AATGCGAGTGGTCTTCCATC
**C/EBPα**	TGAAGGAACTTGAAGCACAA	TCAGAGCAAAACCAAAACAA
**FAS**	GAGGGAAATCCGACAGTTGA	GACTCCAACAGAGCCTGAGC
**ap2**	TCACCTGGAAGACAGCTCCT	AATCCCCATTTACGCTGATG
**ATGL**	CCAACCTTTGTGCCCCTTAA	ATTCTCTTGGTGCCCATGTAGTAGCCCG

### Western blot analysis

Protein analysis was performed using the western blot (WB) method. 3T3-L1 adipocyte proteins were extracted using a protein extraction kit (Nanjing Jiancheng Bioengineering Institute, China) for the treated and untreated mature adipocytes. The protein extracts were obtained using centrifugation (15,000 r/min, 10 min) and stored at −80°C. The protein concentration of the extracts was determined by the BCA kit (Beyotime Biotechnology, Shanghai, China). Protein electrophoresis was performed with a 10% SDS-polyacrylamide electrophoresis system, 50 V of concentrated gel, and 120 V of separator gel, and bromophenol blue as the tracer dye. After SDS-polyacrylamide electrophoresis, the proteins were transferred to the PVDF membrane. An immunological blot was detected using an ECL substrate reagent. PPARγ, C/EBP-α, FAS, FABP4, and ATGL antibodies were used as the first antibody, and the conjugated Goat anti-Rabbit IgG (H + L) was the second antibody.

### UPLC-QTOF-MS/MS analysis

The HBP extract was qualitatively analyzed using ultra-performance liquid chromatography quadrupole time-of-flight mass spectrometry (UPLC-QTOF-MS/MS) (Agilent 1290–6545, Agilent Technologies, USA). The column used in the method was poroshell 120 column (2.1 × 100 mm, 2.7 μm, Agilent Technology, USA). The column temperature was set to 45°C. The mobile phase consisted of formic acid in water (0.1%) as solvent A and methanol as solvent B using gradient elution. The injection volume of the prepared sample was 2 μL and the flow rate was 0.3 mL/min. The solvent gradient was 0 to 1.5 min, 5% B; 5 min, 15% B; 9 min, 25% B; 16 min, 40% B; 22 min, 55% B; 28 to 30 min, 95% B; and 31 min, 5% B. Mass spectrometry was performed using electrospray source in negative ion mode with full scan, auto MS/MS mode. The capillary voltage was 3,500 V, nozzle voltage was 500 V, drying-gas temperature was 260°C, and sheath gas temperature was 360°C. Mass spectrometry data were analyzed with the MassHunter Workstation Software (Quantitative Analysis B.07.00, PCDL Manager B.07.00, and Molecular Structure Correlator, Agilent Technology, USA).

### Statistical analyses

All the results were expressed as mean ± standard deviation (SD), and the number of measurements (*n* = 3) characterized the individual experiments. The significant associations were determined using the one-way analysis of variance (ANOVA) followed by Duncan's multiple tests. A *p* < 0.05 was considered statistically significant.

## Results

### Phenolic composition of HBP extract

The total ion chromatography of HBP extract was presented in Supplementary Figure 1. Sixty-eight compounds were identified by matching retention times (RT), m/z values, MS/MS fragments with compounds from Metlin database (https://metlin.scripps.edu), TCM database (http://tcm.cmu.edu.tw/), PubChem (https://pubchem.ncbi.nlm.nih.gov), and the database based on standards. These compounds included 5 falvones (compounds **44, 53, 59, 61**, and **65**), 1 isoflavones (compound **49**), 4 flavanones (compounds **13, 15, 19**, and **64**), 2 flavans (compounds **4** and **23**), 7 flavanols (compounds **5, 8, 11, 14, 18, 24**, and **29**), 18 flavonols (compounds **7, 17, 20, 27, 28, 32, 34, 36, 37, 38, 41, 43, 45, 46, 51, 60, 66**, and **68**), 16 flavonoid glycosides (compounds **1, 16, 25, 35, 39, 47, 48, 50, 52, 54, 55, 56, 57, 58, 62**, and **63**), 3 anthocyanidins (compounds **26, 33**, and **67**), 1 neoflavonoids (compound **40**), 1 tetracenomycin (compound **6**), 1 dihydroxy-phenylacetic acid (compound **2**), 3 dihydroxy-benzoic acids (compounds **3, 21** and **22**), 1 benzodioxoles (compound **9**), 1 hydroxybenzaldehyde (compound **10**), 1 coumaric acids (compound **12**), 2 hydroxycinnamic acids (compounds **30** and **31**), and 1 lignan (compound **41**) (Table 2). In addition, the parent and daughter ion information in UPLC-QTOF-MS/MS was shown in Supplementary Figure 2.

**Table 2:** Phenolic compounds identified in HBP extract by UPLC-QTOF-MS/MS.

**Number**	**RT (min)**	**Molecular formula**	**[M–H]^−^(*m/z*)**	**Error (ppm)**	**MS/MS fragments (*m/z*)**	**Tentative identification**	**Database**			
							**Metlin**	**TCM**	**PubChem**	**standards**
1	6.04	C_30_H_26_O_14_	609.1272	−3.6	177.0196, 457.0784, 89.0246, 125.0248	6”–O–Caffeoylastragalin	√		√	
2	6.35	C_8_H_8_O_4_	167.0352	−1.3	123.0453, 149.0238, 105.0337	Homogentisic acid			√	√
3	6.92	C_7_H_6_O_4_	153.02	−4.4	121.0283, 109.03, 136.0161	Protocatechuic acid			√	√
4	7.08	C_15_H_14_O_7_	305.0682	−5	137.0246, 125.0249, 165.0194	(–)–epigallocatechin			√	
5	7.34	C_27_H_34_O_16_	613.1802	−4.6	451.1261, 289.0725, 137.0245	Leiocarposide	√		√	
6	7.61	C_22_H_16_O_8_	407.079	−4.3	285.0404, 297.0404, 381.0988	Tetracenomycin B2	√		√	
7	7.97	C_30_H_26_O_13_	593.1324	−3.9	441.0838, 137.0245, 125.0246, 425.0892	Tiliroside		√	√	
8	8.24	C_45_H_38_O_19_	881.1967	−3.7	713.1522, 125.0246, 289.0719	Gallocatechin–(4alpha–>8)–catechin–(4alpha–>8)–catechin	√		√	√
9	8.47	C_7_H_6_O_3_	137.0248	−2.8	109.0294, 92.0273, 119.0138	Sesamol		√	√	
10	10.95	C_7_H_6_O_2_	121.0297	−1.6	92.027, 93.0344, 95.0126	p–Hydroxybenzaldehyde		√	√	√
11	11.25	C_30_H_26_O_12_	577.1375	−4.1	137.0239, 125.025, 425.0896	Procyanidin B2			√	
12	11.54	C_14_H_20_N_4_O_2_	275.1522	−3.1	233.1302, 119.0507, 258.1244	p–Coumaroylagmatine	√		√	
13	11.75	C_27_H_32_O_15_	595.1685	−2.8	167.0358, 137.0243, 493.1398	Neoeriocitrin		√	√	√
14	12.11	C_21_H_22_O_12_	465.1056	−3.8	167.0353, 125.0244, 329.0884	Epicatechin 3'–O–glucuronide			√	
15	12.11	C_21_H_22_O_12_	465.1056	−3.8	303.0507, 167.0353, 125.0244	Plantagoside		√	√	
16	12.11	C_21_H_22_O_12_	465.1056	−3.8	303.0507, 285.041, 167.0353	Glucodistylin	√		√	
17	12.66	C_16_H_14_O_7_	317.068	−4.2	125.0245, 149.061, 179.0352, 273.078	Dihydroisorhamnetin	√		√	
18	12.77	C_22_H_24_O_12_	479.1218	−4.8	447.0948, 285.0404, 299.056	4'–O–Methyl–(–)–epicatechin 3'–O–glucuronide	√		√	√
19	13.06	C_21_H_20_O_11_	447.0954	−4.7	299.0562, 89.0244, 125.025	Naringenin 7–O–glucuronide			√	
20	13.06	C_21_H_20_O_11_	447.0954	−4.7	285.0406, 299.0562	Astragalin	√		√	√
21	13.20	C_8_H_8_O_4_	167.0355	−3.1	152.0117, 121.0296, 135.0078	Vanillic acid			√	
22	13.30	C_13_H_18_O_5_	253.109	−3.4	191.1084, 209.1187, 151.0762	Hostmaniane	√		√	
23	13.80	C_45_H_38_O_18_	865.1989	−0.4	245.0443, 577.1366, 713.151	Cinnamtannin A1	√		√	
24	14.45	C_23_H_22_O_12_	489.1055	−3.4	285.0402, 269.0455, 299.0567, 461.1083	6”–O–Acetylastragalin	√		√	
25	14.63	C_23_H_24_O_13_	507.1167	−4.5	429.0831, 328.0586, 371.0988, 461.1085	4',8–Dimethylgossypetin 3–glucoside	√		√	
26	15.77	C_27_H_30_O_15_	593.154	−4.7	431.0987, 311.0562, 469.0764	6–Hydroxypelargonidin 3–rutinoside	√		√	
27	15.91	C_23_H_24_O_13_	507.1166	−4.3	299.0564, 285.0406, 429.0843	Tomentin 6–galactoside	√		√	
28	16.07	C_25_H_26_O_15_	565.1216	−3	285.0406, 125.0243, 529.0966	Quercetin 3–xylosyl–(1–>2)–alpha–L–arabinofuranoside	√		√	
29	16.19	C_27_H_34_O_17_	629.1755	−5	587.1633, 285.0408, 563.1424	Leucodelphinidin 3–O–(beta–D–glucopyranosyl–(1–>4)–alpha–L–rhamnopyranoside)	√		√	√
30	16.21	C_10_H_10_O_4_	193.0514	−4	178.0273, 149.0609	Ferulic acid (3–(4–Hydroxy−3–methoxyphenyl)−2–propenoic acid)			√	√
31	16.21	C_10_H_10_O_4_	193.0514	−4	178.0273, 149.0609	Isoferulic acid			√	
32	16.33	C_23_H_22_O_12_	489.1059	−4.2	89.0243, 285.0405, 299.0555	Kaempferol 3–(6–acetylgalactoside)	√		√	
33	16.53	C_31_H_32_O_18_	691.1513	0.4	529.0998, 563.1423, 623.1643	3,5–di–O–(beta–Glucopyranosyl) pelargonidin 6”–O−4, 6”'–O−1–cyclic malate	√		√	√
34	16.57	C_34_H_42_O_20_	769.2224	−3.6	623.1625, 461.1107	Typhaneoside		√	√	
35	16.57	C_34_H_42_O_20_	769.2224	−3.6	623.1625, 461.1107	Isorhamnetin 3–rutinoside 4'–rhamnoside	√		√	√
36	17.10	C_15_H_10_O_7_	301.0362	−2.7	257.0459,149.0602	Quercetin (3 3' 4' 5 7–pentahydroxyflavone)			√	
37	17.10	C_15_H_10_O_7_	301.0362	−2.7	257.0459, 149.0602, 228.0714	6–Hydroxykaempferol	√		√	
38	17.26	C_27_H_30_O_15_	593.1534	−3.7	383.0779, 503.1204, 473.1105	Nicotiflorin		√	√	
39	17.26	C_27_H_30_O_15_	593.1534	−3.7	383.0779, 299.0552, 503.1204	Graveobioside B	√		√	
40	17.26	C_27_H_30_O_15_	593.1534	−3.7	299.0552, 383.0779, 503.1204	5,3',4'–Trihydroxy−7–methoxy−4–phenylcoumarin 5–O–xylosyl–(1–>6)–glucoside	√		√	
41	17.29	C_29_H_34_O_18_	669.1698	−3.8	603.1703, 623.1642	Limocitrin 3,7–diglucoside	√		√	
42	17.98	C_28_H_38_O_13_	581.2266	−4.5	373.1306, 179.056, 401.1621	(+)–Lyoniresinol 9'–O–glucoside	√		√	√
43	18.47	C_28_H_32_O_16_	623.1637	−3.1	341.0673, 285.04, 443.1	Narcissoside		√	√	
44	18.47	C_28_H_32_O_16_	623.1637	−3.1	341.0673, 285.04, 443.1	Pasternoside	√		√	√
45	18.88	C_15_H_10_O_6_	285.0411	−2.2	149.0242, 109.0297, 257.0461	Kaempferol (3 4' 5 7–tetrahydroxyflavone)	√		√	√
46	18.88	C_15_H_10_O_6_	285.0411	−2.2	133.0297, 121.0296, 257.0461	Fisetin		√	√	
47	19.21	C_26_H_32_O_14_	567.1737	−3.1	549.1624, 489.1395	Cis–Mulberroside A	√		√	
48	19.23	C_23_H_22_O_13_	505.1008	−4	300.0279, 461.0728, 447.095	Glyphoside	√		√	√
49	19.30	C_22_H_22_O_11_	461.1111	−4.7	298.0487, 285.0409, 341.0677, 371.0787	Tectoridin	√		√	
50	19.56	C_33_H_40_O_19_	739.2109	−2.4	269.0458, 161.0247	Clitorin		√	√	
51	19.56	C_33_H_40_O_19_	739.2109	−2.4	269.0458, 161.0247	Kaempferol 3–(2”–rhamnosylrutinoside)	√		√	
52	19.93	C_34_H_42_O_20_	769.2219	−2.9	299.0567, 721.1999	Isorhamnetin 3–O–[a–L–rhamnopyranosyl–(1–>3)–a–L–rhamnopyranosyl–(1–>6)–b–D–glucopyranoside]	√		√	
53	20.44	C_27_H_30_O_14_	577.1581	−3.2	269.0459, 72.9932	Rhoifolin		√	√	
54	20.44	C_27_H_30_O_14_	577.1581	−3.2	269.0459, 72.9932	Galangin 3–[galactosyl–(1–>4)–rhamnoside]	√		√	
55	20.56	C_38_H_40_O_19_	799.2128	−4.6	101.0246, 207.066, 461.1105	6”'–O–Sinapoylsaponarin	√		√	√
56	20.64	C_28_H_32_O_16_	623.164	−3.6	315.0516	Isorhamnetin−3–O–neohespeidoside		√	√	
57	20.64	C_28_H_32_O_16_	623.164	−3.6	315.0516	Isorham netin 3–O–[b–D–glucopyranosyl–(1–>2)–a–L–rhamnop60yranoside]	√		√	√
58	20.99	C_28_H_32_O_15_	607.168	−1.9	299.0568	Spinosin		√	√	
59	20.99	C_28_H_32_O_15_	607.168	−1.9	299.0568	Diosmin	√		√	√
60	21.14	C_21_H_20_O_11_	447.0954	−4.7	285.0409, 112.9857	Quercitrin		√	√	
61	21.78	C_22_H_20_O_12_	475.09	−3.8	284.0331, 85.0297, 299.0566, 133.0245	Scutellarin methylester		√	√	
62	21.78	C_22_H_20_O_12_	475.09	−3.8	284.0331, 299.0566	Diosmetin 7–O–beta–D–glucuronopyranos66ide	√		√	
63	21.86	C_23_H_22_O_13_	505.1007	−3.8	329.0677, 314.0438, 314.0438	Tricin 7–glucuronoside	√		√	√
64	21.96	C_16_H_14_O_6_	301.0726	−2.8	286.0487, 269.0459	Hesperetin			√	
65	22.12	C_22_H_22_O_11_	461.1112	−4.9	299.0565, 113.0246, 285.0395	Kaempferide 3–galactoside	√		√	√
66	24.90	C_16_H_12_O_7_	315.0522	−3.7	300.0279, 301.0314	Isorhamnetin		√	√	
67	24.90	C_16_H_12_O_7_	315.0522	−3.7	300.0279, 301.0314	Petunidin	√		√	
68	24.90	C_16_H_12_O_7_	315.0522	−3.7	300.0279, 301.0314	Junipegenin A	√		√	√

### Total phenol content and enzyme-inhibitory activity

**Figure 1:**
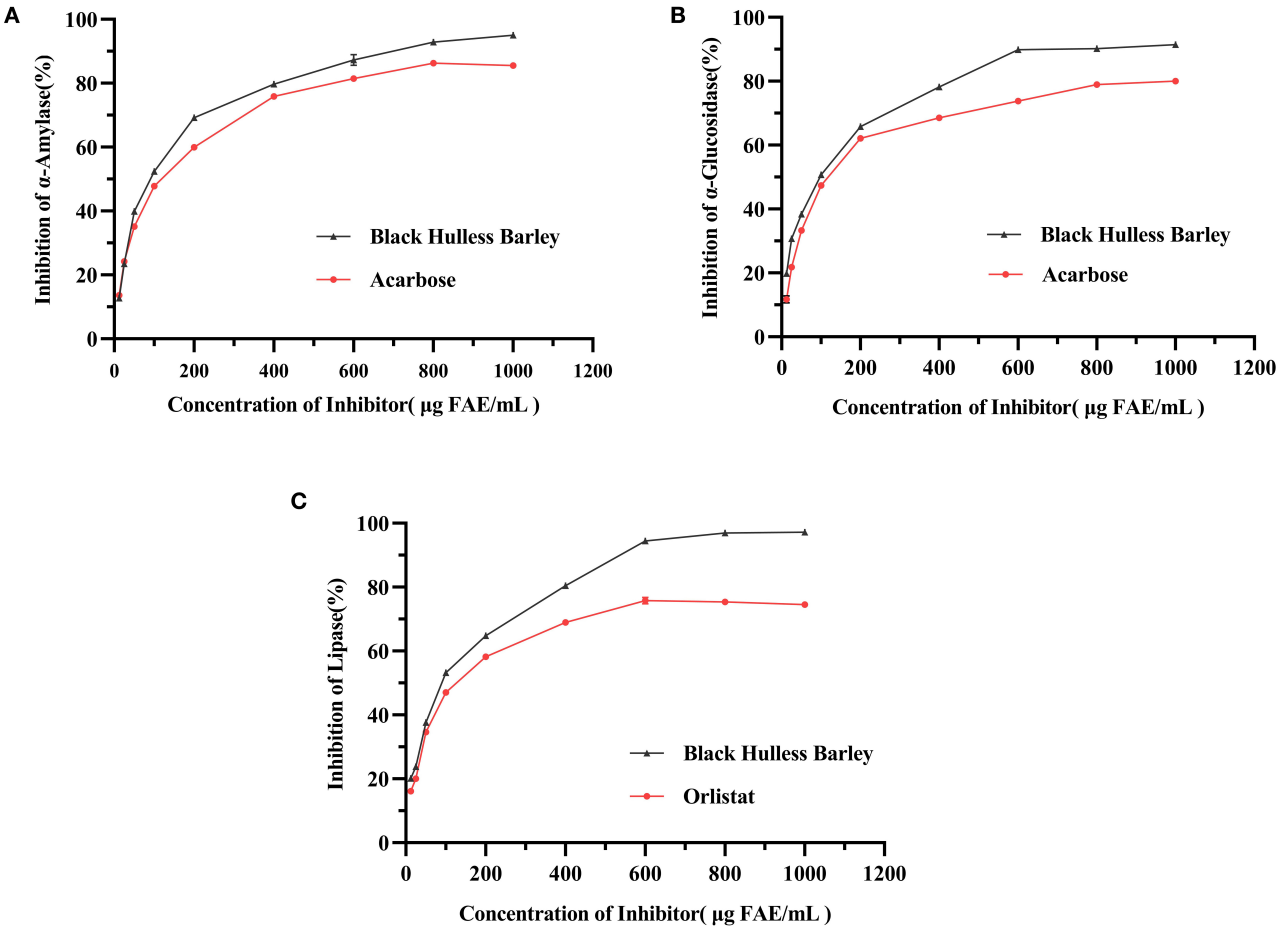
The inhibitory activities of HBP extract on α-amylase **(A)**, α-glucosidase **(B)**, and lipase **(C)**. The enzyme samples were treated with different concentrations of HBP extract, and the positive control groups were treated with acarbose (for α-amylase and α-GLU) and orlistat (for lipase). Data were expressed as mean measurements (*n* = 3).

The TPC of the HBP extract was 1016.16 ± 3.81 μg FAE/mL. The inhibitory activities of HBP extract were determined using different concentrations (12.5–1000 μg FAE/mL) against α-amylase, α-GLU, and lipase. The extracts inhibited α-amylase, α-GLU, and lipase in a dose-dependent manner (Figures 1A–C). For α-amylase, α-GLU, and lipase inhibition, HBP extract had similar characteristics as the positive control. Moreover, the inhibitory activities of α-amylase, α-GLU, and lipase were better than positive drugs at similar concentrations.

The half-maximal inhibitory concentration (IC_50_) characterized the enzyme inhibition effect. When the concentration of the inhibitor was elevated from 12.5 to 1,000 μg FAE/mL, the IC_50_ values of the HBP extract against α-amylase, α-GLU, and lipase were 84.84, 83.91, and 80.94 μg FAE/mL, respectively (Table 3). These results were better than acarbose for α-amylase, α-GLU, and orlistat for lipase. Therefore, HBP extract exhibited a better inhibitory effect against the three enzymes than the positive control.

**Table 3:** α-Amylase, α-GLU, and Lipase Inhibitory activity by HBP extract.

**Enzyme**	**IC**_**50**_ **(mean** ±**SD in** μ**g FAE/ ml)**	
	**HBP extract**	**Reference inhibitor**
α-amylase	84.84 ± 0.79^a^	111.83 ± 0.91 (Acarbose)^b^
α-GLU	83.91 ± 1.52^a^	134.83 ± 4.27 (Acarbose)^b^
Lipase	80.94 ± 0.33^a^	143.10 ± 3.18 (Orlistat)^b^

*Different superscript letters (a, b) in a row indicate a significant difference between means (p <0.05)*.

### Cytotoxicity of HBP extract on 3T3-L1 preadipocytes and mature adipocytes

**Figure 2:**
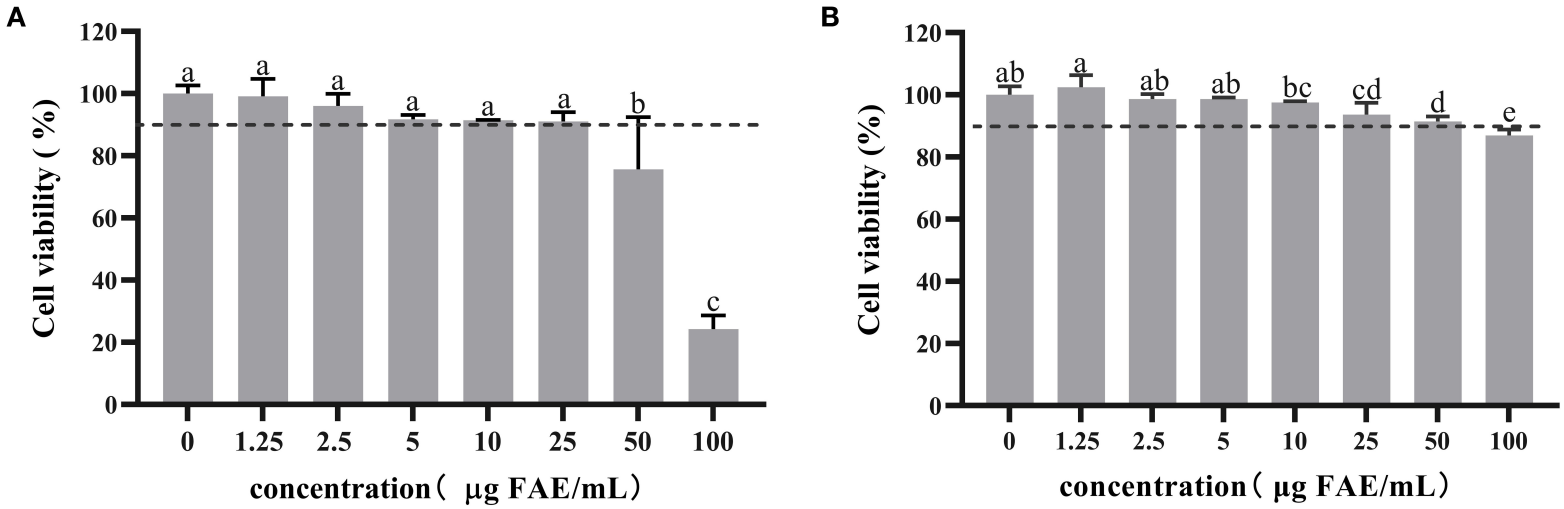
Cytotoxicity of HBP extract on 3T3-L1 preadipocytes and mature adipocytes. **(A)** 3T3-L1 preadipocytes were differentiated with or without HBP extract for 24 h. **(B)** 3T3-L1 mature adipocytes were treated or untreated with HBP extract for 24 h. Cell viability was measured by MTT assay. Different letters indicate significant differences at *p* < 0.05.

The concentration is considered non-toxic to cells when cell viability is > 90%. Therefore, 3T3-L1 preadipocytes and mature adipocytes were treated with different concentrations of HBP extract to determine its effect on cell viability. Initially, the cytotoxicity was examined by treating the cells with 1.25 to 100 μg FAE/mL of the HBP extract for 24 h. MTT analysis of the preadipocytes revealed that the cell viability was 75.6% at 50 μg FAE/mL HBP extract concentration. The observed viability was less than the 90% cell viability threshold and affected the viability of 3T3-L1 preadipocytes (Figure 2A). However, the MTT assay of the 3T3-L1 mature adipocytes showed that the cell survival rate was 86.9% at a 100 μg FAE/mL concentration, indicating toxicity (Figure 2B). Next, we selected three non-toxic HBP extract concentrations (5, 10, and 25 μg FAE/mL) to maintain an appropriate number of cells for manipulation for follow-up experiments based on the above results.

### HBP extract inhibits adipogenesis and TG accumulation

**Figure 3:**
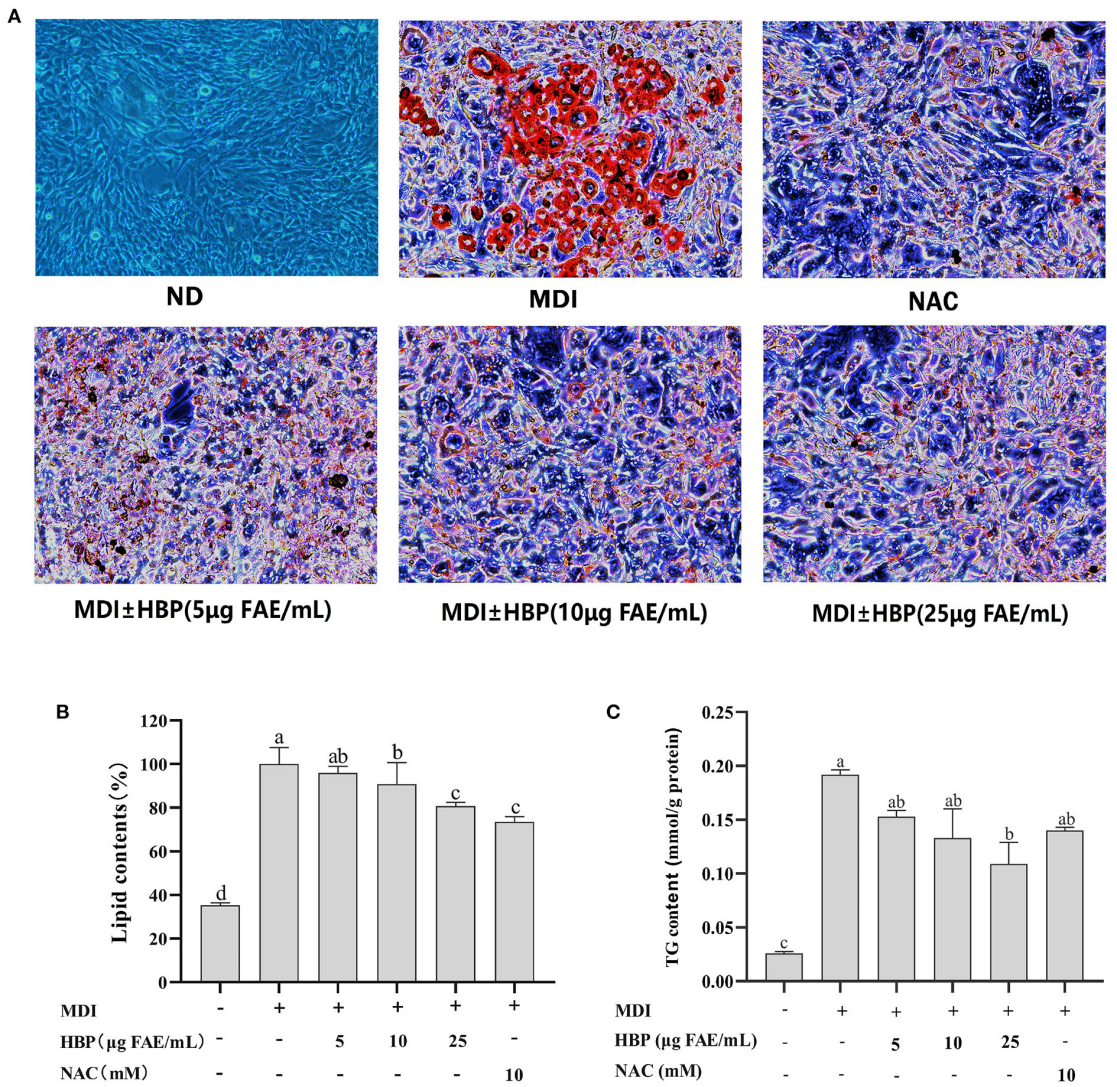
Effects of HBP extract on adipocyte differentiation in 3T3-L1 cells. **(A)** 3T3-L1 preadipocytes were cultured in a differentiation medium containing 5, 10, and 25μg FAE/mL for 48 h. The cells were then stained with oil red O staining solution. “ND” means that cells were cultured in a normal medium without HBP extract. “MDI” means that cells were cultured in a differentiation medium. NAC was used as a positive control group. **(B)** The cells stained with Oil red O were subjected to quantitative analysis of the intracellular lipid accumulation. **(C)** HBP extract inhibited TG accumulation in 3T3-L1 adipocytes. All values are presented as the mean ± SD of three experiments performed in triplicate. Different letters indicate significant differences at *p* < 0.05.

The effect of HBP extract on the amount of fat accumulated in adipocytes was investigated through staining and quantification of the intracellular lipids stained with Oil Red O and quantified. Undifferentiated preadipocytes were used as the control group (ND). In contrast, the differentiated culture group with the drug was the positive control group (NAC). The microscope images (Figure 3A) showed that HBP extract exhibited an anti-differentiation effect similar to the NAC group by reducing the lipid content accumulation. Moreover, the number and size of lipid droplets decreased with the increase in HBP extract dose.

Furthermore, the lipid quantification assay confirmed the decrease of fat accumulation during the dose-dependent adipocyte maturation (Figure 3B). The observed results were corroborated under the microscope. Moreover, lipid contents of 10 and 25 μg FAE/mL HBP extract-treated cells decreased by 9.128% and 9.30%, respectively. When the concentration was 25 μg FAE/mL, there was no significant difference (*P* > 0.05) compared to the positive control (NAC), indicating the efficient inhibition of the differentiated 3T3-L1 cells by the particular concentration of the HBP extract. These results revealed that HBP extract could inhibit the differentiation of 3T3-L1 preadipocytes and intracellular lipid accumulation.

Furthermore, adipogenesis reduction can be responded to by observing the delipidating effect. Thus, we quantified the intracellular TG contents. The effect of HBP extract on TG levels is shown in Figure 3C. It was observed that 5, 10, and 25 μg FAE/mL of the HBP extract reduced the TG levels within 3T3-L1 adipocytes by 0.153, 0.132, and 0.109 mmol/g protein, respectively. Therefore, the results indicated that intracellular TG levels were significantly reduced (*P* < 0.05) compared to the MDI group after HBP extract treatment. In particular, when the concentration was 25 μg FAE/mL, HBP extract exhibited a more substantial inhibitory effect than the positive control (NAC).

### HBP extract arrests cell cycle progression in 3T3-L1 preadipocytes

**Figure 4:**
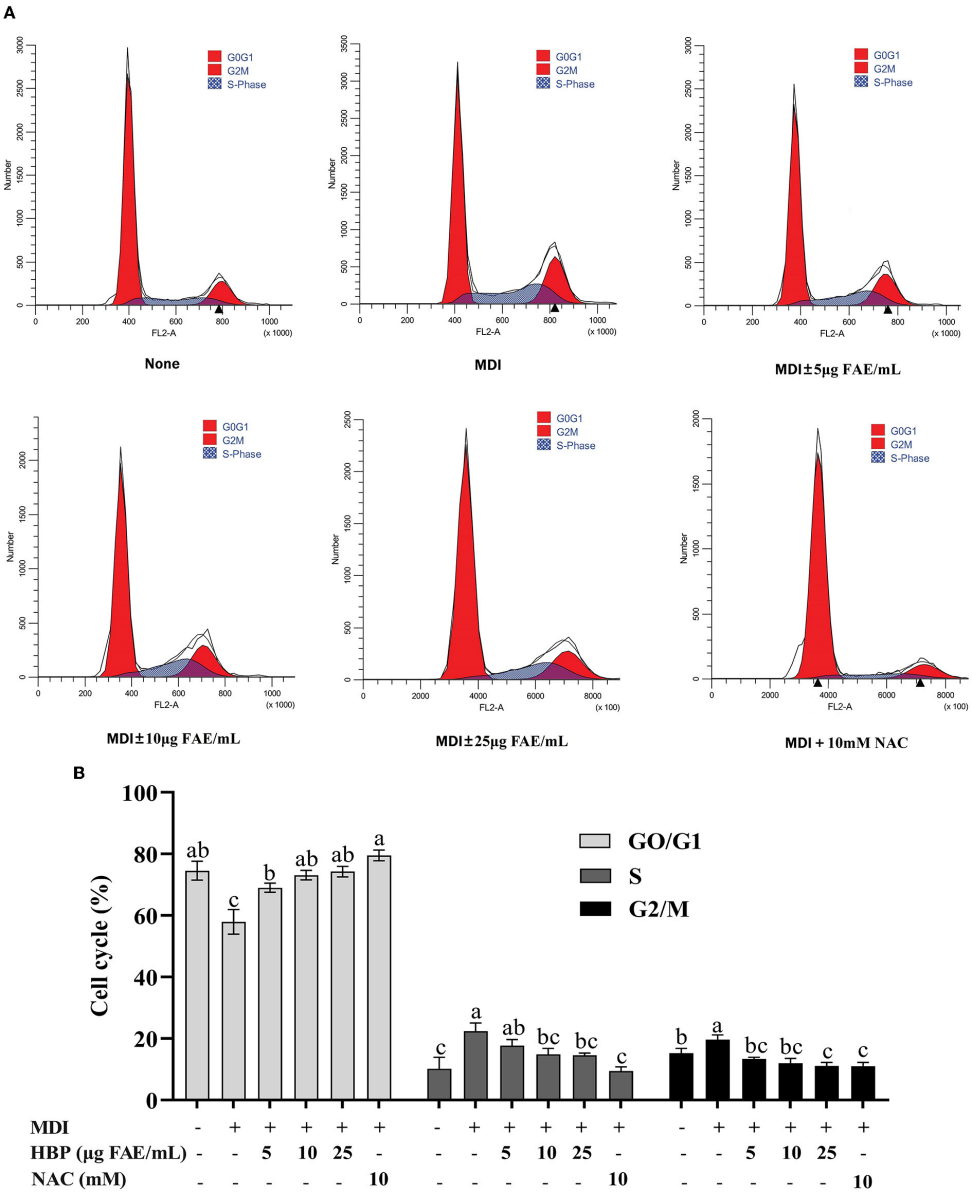
Regulatory role of HBP extract on cell cycle progression in 3T3-L1 adipocytes. **(A)** 3T3-L1 preadipocytes were incubated in MDI with or without HBP extract for 24 h. The cells were harvested and stained with PI, and then the cell cycle was analyzed using flow cytometry. **(B)** Quantification of the cell cycle stage distribution. The values were expressed as the mean ± SD, *n* = 3.

Inhibition of cell growth may be mediated through the arrest of the cell cycle effect. Therefore, flow cytometry was used to analyze the distribution of the cell cycle. It was observed that the cell population increased in the G0/G1 phase and decreased in the S phase and G2/M phase with the increasing concentration of HBP extract (Figures 4A,B). At 25 μg FAE/mL HBP extract concentration, the cell population decreased by 2.19 and 2.83% in the S and G2/M phases, respectively, compared to the MDI group. However, the cell population in the NAC group was significantly reduced in the S (19.72 to 8.43%) and the G2 phases (18.83 to 10.04%) (compared to the MDI group). Treatment of 3T3-L1 preadipocytes with HBP extract exhibited delayed entry of cells into the S and G2/M phases. Therefore, flow cytometry analysis revealed that HBP extract blocks the differentiation of 3T3-L1 preadipocytes in the early stages.

### HBP extract ameliorates cell oxidative stress

**Figure 5:**
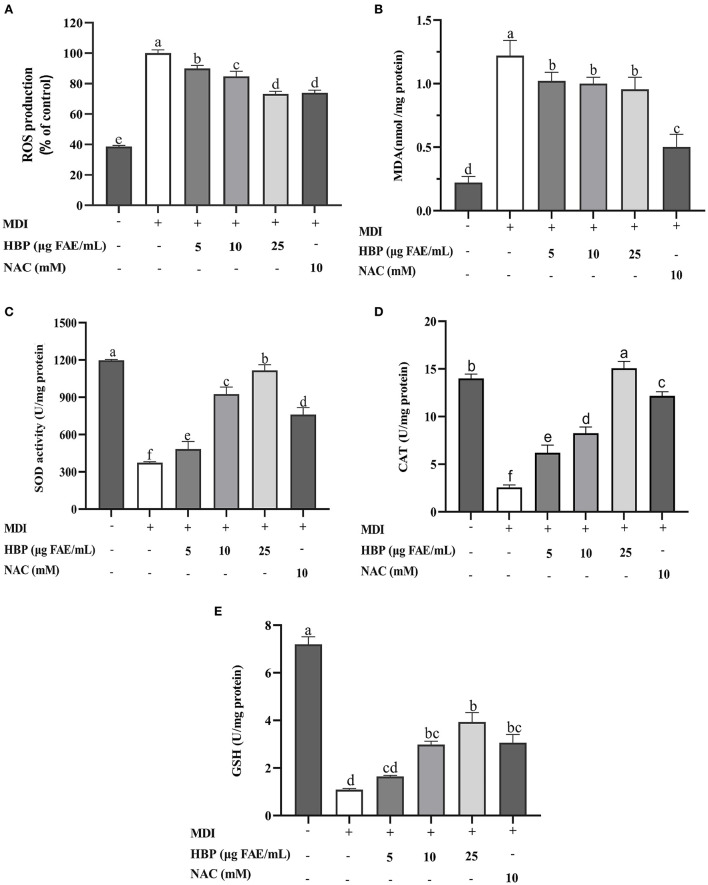
Effect of HBP extract on antioxidant activity of 3T3-L1 adipocytes. **(A)** Reactive oxygen species (ROS). **(B)** Malondialdehyde (MDA). **(C)** Superoxide dismutase (SOD), **(D)** Catalase (CAT). **(E)** Glutathione (GSH), and Malondialdehyde (MDA). NAC was the positive control group, and different lowercase letters indicate significant differences (*p* < 0.05).

The addition of HBP extract to the culture medium during adipocyte maturation resulted in a dose-dependent decrease in ROS production. The 25 μg FAE/mL of HBP extract concentration resulted in a 26.7% reduction in the ROS production compared to the MDI group (Figure 5A). However, the cells in the control group were not differentiated, with low ROS levels. Moreover, there was no significant difference between the sample group treated with 25 μg FAE/mL of the HBP extract and the NAC group (*P* > 0.05).

Next, we measured the effect of HBP extract on the malondialdehyde (MDA) levels of the 3T3-L1 adipocytes. Figure 5B shows that the contents of MDA in the sample groups (treated with various concentrations of the HBP extract) were significantly elevated (*P* < 0.05) compared to the control group (MDI). However, there was a non-significant decrease in the contents of intracellular MDA in the sample groups (*P* > 0.05), which were higher than the positive control group (NAC).

Superoxide dismutase (SOD) and Catalase (CAT) are critical cellular antioxidant enzymes. Our results indicated that the activities of SOD and CAT significantly increased (*P* < 0.05) in a concentration-dependent manner when treated with variable concentrations of the HBP extract. When the cells were treated with 5, 10, and 25 μg FAE/mL of HBP extract, the SOD activities improved by 17.5%, 125%, and 172%, while the CAT activities increased by 141.6%, 220.8%, and 485.6%, respectively (Figures 5C,D). Moreover, intracellular SOD and CAT activities were higher than the positive control (NAC) at 25 μg FAE/mL of HBP extract concentration.

Finally, the contents of intracellular GSH were determined. Figure 5E indicates that the GSH levels significantly increased (*P* < 0.05) when the cells are treated with the HBP extract. At 10 and 25 μg FAE/mL, the GSH contents of the incubated sample group were 2.985 U/mg protein and 3.938 U/mg protein, respectively. Thus, HBP extract had good antioxidative stress properties against 3T3-L1 adipocytes.

### Effect of HBP extract on the expression of adipogenesis-associated genes and proteins in 3T3-L1 cells

**Figure 6:**
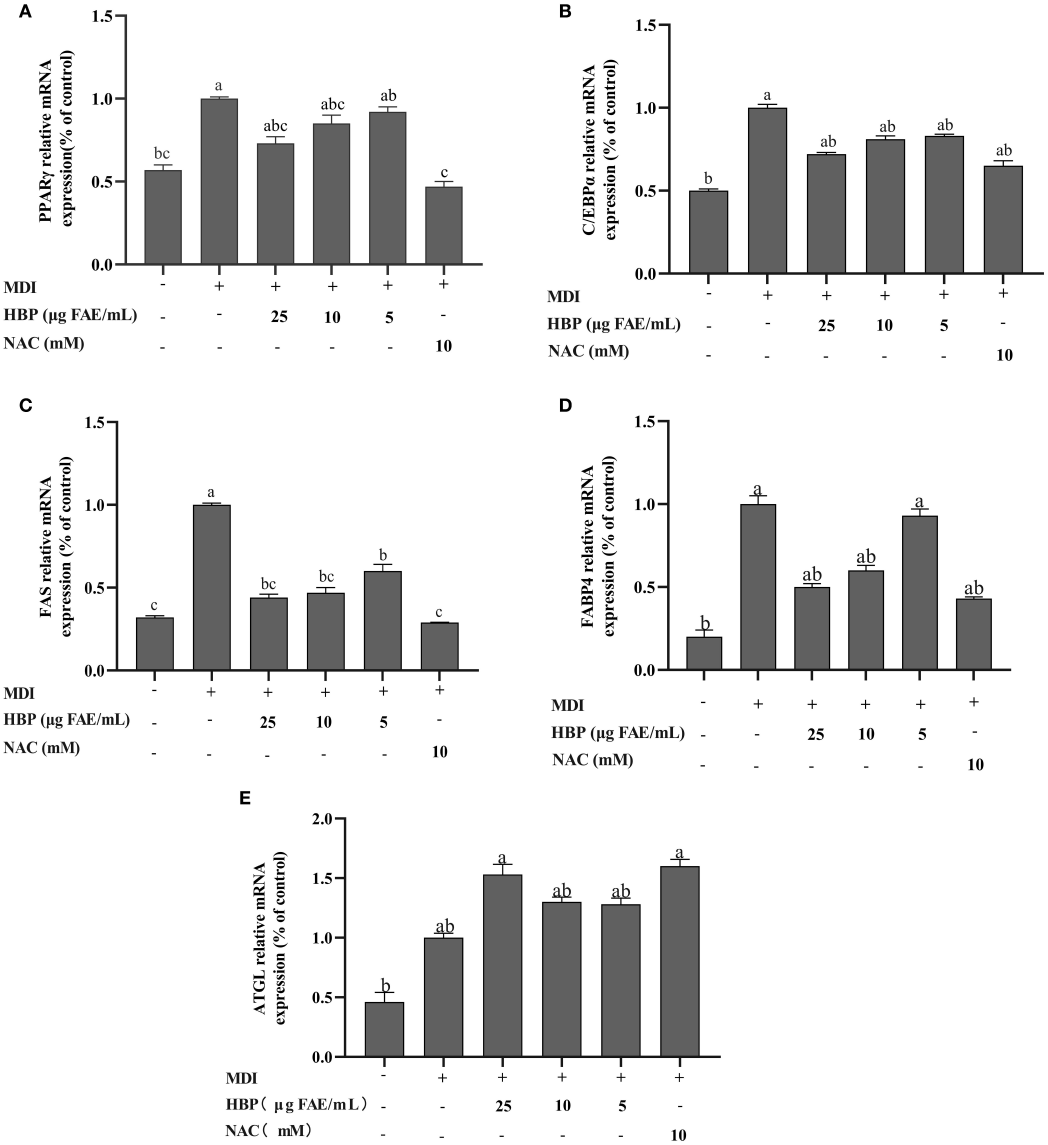
Effect of HBP extract on the mRNA expression of adipogenic genes in 3T3-L1 adipocytes. The transcript levels of **(A)** PPARγ. **(B)** C/EBPα. **(C)** FAS. **(D)** FABP4, and **(E)** ATGL were measured by RT-qPCR. The data are presented as the mean ± SD, *n* = 3. Different lowercase letters denote significant differences (*P* < 0.05).

**Figure 7:**
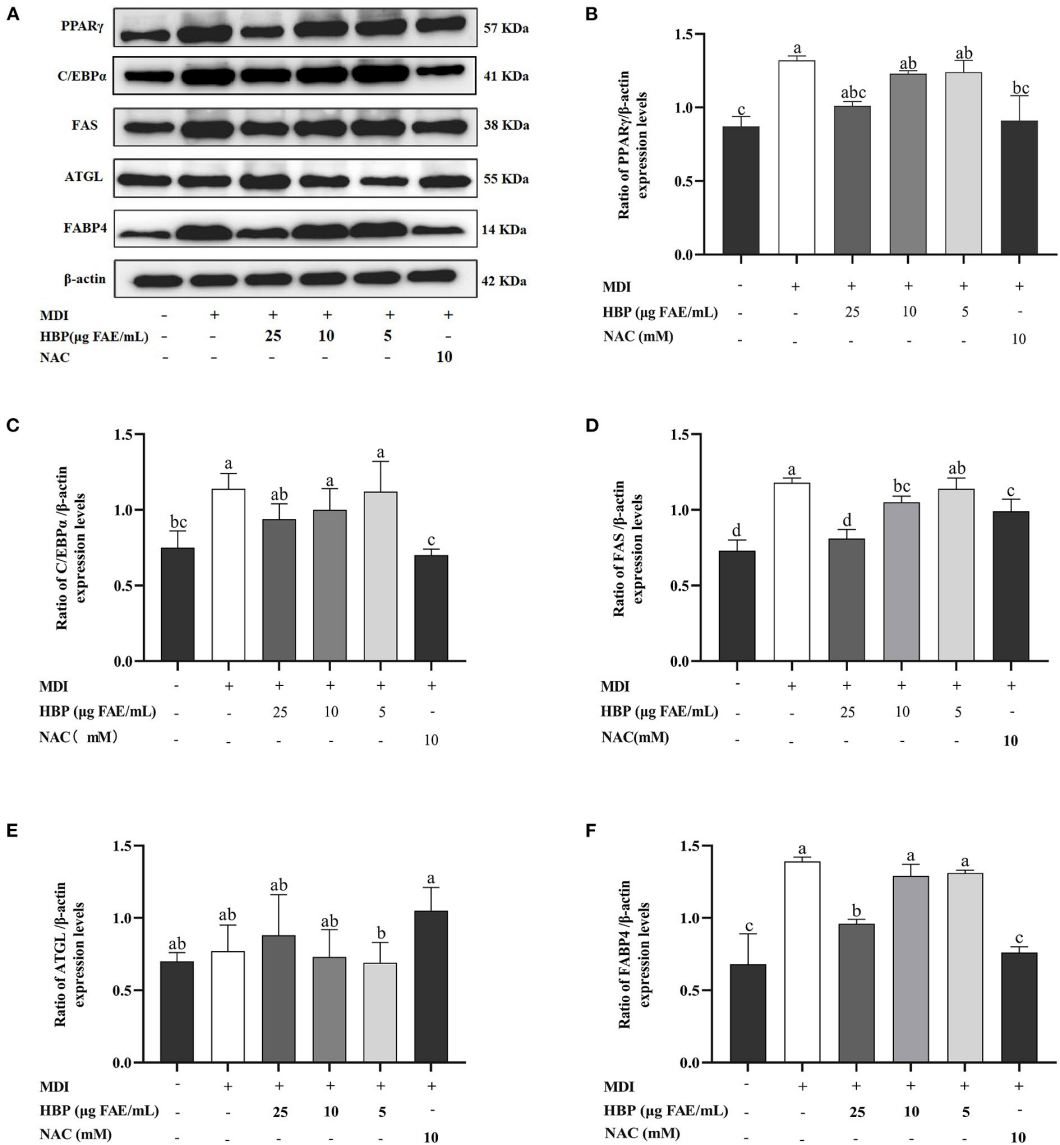
Effect of HBP extract on the expression of adipogenesis-associated genes in 3T3-L1 adipocytes. **(A)** The protein expression of PPARγ, C/EBPα, FAS, ATGL, FABP4, and β-actin and **(B-F)** the relative protein levels were measured by Western blot analysis. Data are expressed as mean ± SD, *n*=3, and different lowercase letters indicate significant differences (*p* < 0.05).

Adipocyte differentiation involves the expression of many adipogenic and lipid metabolism-related genes and proteins. HBP extract suppressed PPARγ, C/EBPα, FAS, and FABP4 proteins and gene expressions in 3T3-L1 cells in a concentration-dependent manner (Figures 6A–D, 7B–D,F). HBP extract also significantly increased (P <0.05) ATGL protein expression and enhanced the expression of the ATGL gene compared to the control group (Figures 6E, 7E). These results validated the protein blot analysis in Figure 7A. Notably, the β-actin level, an internal control, was not affected by the HBP extract. Besides, the uncropped western blotting images are presented in the online Supplementary material (see Supplementary Table 1).

## Discussion

Obesity is a public health problem prevalent in both developed and developing nations (38). Obesity usually occurs due to the hyperplasia of adipose tissue and lipid accumulation (12). Recently, enzymatic activity inhibition related to fat and carbohydrate metabolism, adipocyte differentiation, and related gene expression is a potential therapeutic strategy against obesity. Moreover, an increased anti-obesity application of cereal polyphenols has been discovered in recent years. Previous studies have investigated the antioxidant activity of phenolic compounds derived from hulless barley (39). The murine 3T3-L1 preadipocyte cell line was selected as a primary model for shedding further light on the potential lipid-lowering effects of the HBP extract. The present study demonstrated that HBP extract could prevent obesity through various inhibition assays against metabolically essential enzymes. Moreover, HBP extract inhibited the differentiation of 3T3-L1 preadipocytes. Furthermore, HBP extract also repressed intracellular lipid accumulation by diminishing oxidative stress and regulating adipogenic gene expression.

The TPC of HBP extract was quantified by the Folin-Ciocalteu method. Our results showed that the TPC of HBP extract was 1016.16 ± 3.81 μg FAE/mL. A previous study showed that the TPC of 12 blue highland barley was 336.21 to 453.94 mg GAE/100 g DW (40). The inconsistent results may be due to the different methods for estimation of TPC. In addition, phenolic compounds were identified by comparing their parent and daughter ions information and molecular masses, molecular formulae, RT, and m/z values with databases. A total of 68 compounds were identified in the HPB extract. After detecting by UPLC-QTOF-MS/MS, we found that both HPB extract and fermented hulless black barley (41) contained p-coumaroylagmatine. In addition, we found that epicatechin 3'-O-glucuronide in HBP extract was a derivative of (-)-epicatechin in fermented hulless black barley. This may be due to the breakage of molecular bonds between phenolic compounds after fermentation.

Studies have shown that extracts rich in polyphenols can inhibit the enzyme activities involved in glucose and fat metabolism (such as α-amylase, α-GLU, and lipase) (31, 39). The current study demonstrated the ability of HBP extract to inhibit these enzymes. Additionally, a similar study reported the inhibitory effect of purple maize anthocyanin-rich water extract (PMW) on α-amylase (13). PMW exhibited inhibition against α-amylase with IC_50_ values ranging from 109.5 to 172.7 μg/mL. However, the HBP extract inhibited α-amylase with an IC_50_ value of 84.84 μg FAE/mL (Table 3). Interestingly, the IC_50_ value for the HBP extract in our study was much lower (more effective) than the purple maize phenolic extract. In addition, other studies have utilized the polyphenols from the stem and leaf of lentils to study the inhibition of α-GLU and lipase. The results exhibited that the inhibition of α-GLU and lipase increased with elevated polyphenol concentration (42), consistent with our findings.

Differentiation of adipocytes is associated with the expression of adipogenic genes (43). PPARγ and C/EBPα are critical adipogenic transcription factors regulating adipogenesis (44). In the current study, we observed that HBP extract inhibited the mRNA expression of PPARγ and C/EBPα (Figures 6A,B) and the protein levels of PPARγ and C/EBPα in 3T3-L1 cells (Figures 7B,C). Moreover, the inhibition of PPARγ and C/EBPα led to a decrease in FAS and FABP4 expression. Conversely, ATGL expression was increased after HBP extract treatment since ATGL could specifically hydrolyze triglycerides (TG) and improve lipid accumulation (Figures 6, 7). Therefore, HBP extract is suggested to inhibit the differentiation of preadipocytes by regulating the adipogenic gene expression.

Our results also depicted the inhibitory effect of HBP extract on the accumulation of lipid droplets and TG during adipocyte differentiation (Figure 3). Obesity arises with excessive TG accumulation in adipose tissue (45). In this study, 3T3-L1 cells were treated with HBP extract and found to reduce TG accumulation compared to untreated cells. The ethanolic extracts of Djulis (Chenopodium formosanum, EECF) had effectively suppressed the accumulation of lipids in 3T3-L1 adipocytes by reducing lipid contents and intracellular TG levels (46). Furthermore, similar experiments have established that eating wholegrain rice can reduce total TG levels (47), identical to our data. However, to the best of our knowledge, no study has shown that HBP extract inhibited lipid accumulation.

The number of cells at each cell cycle phase could indirectly reflect adipocyte differentiation (48). Our results depicted that HBP extract delayed the entry of 3T3-L1 adipocytes into the S and G2/M phases by arresting the cell cycle at the G0/G1 junction (Figure 4). In addition, other authors (49) reported that high-polyphenol sorghum bran extracts induced cell cycle arrest in the G1/S phase, with a decrease in the number of cells in the G2 phase. These results support the idea that the HBP extract affects adipocyte differentiation by arresting the cell cycle in the G0/G1 phase.

Previous studies have shown that obesity is a chronic condition arising from oxidative stress due to an imbalance among the active tissue oxygen, ROS, and antioxidants (50). Therefore, regulating oxidative stress could be an effective means to prevent obesity. Previous studies have demonstrated that 7, 8-dihydroxyflavone can reduce intracellular ROS levels through a dose-dependent manner, weaken the activation of the MAPK pathway, and elevate the expression of other antioxidants (51). In addition, many compounds (such as polyphenols) with antioxidant properties have been discovered in grains such as malted wheat (MLT) (52) and pigmented rice (53). In the current study, a large amount of ROS was massively produced during the differentiation of 3T3-L1 preadipocytes. After treatment with variable concentrations of the HBP extract, the ROS contents significantly decreased compared to the control group (Figure 5A), consistent with previous findings. Malondialdehyde (MDA) is a product of cellular lipid oxidation and is widely used as an indicator of oxidative damage to the cell membranes (54). Therefore, reducing intracellular MDA levels can alleviate cell damage. The intracellular MDA levels were significantly decreased post-treatment with HBP extract (Figure 5B) compared to the control group. Besides, superoxide dismutase (SOD) and catalase (CAT) are necessary intracellular antioxidant enzymes that maintain cellular health by controlling free radical levels (55). Glutathione (GSH) is one of the major intracellular non-enzymatic antioxidants, protecting cells against oxidative damage (56). Our results revealed that the activities of SOD and CAT in 3T3-L1 cells were elevated in a concentration-dependent manner after being treated with variable concentrations of the HBP extract (Figures 5C,D). In addition, the intracellular GSH levels also depicted a rising trend after being treated with the HBP extract. Similarly, Hu et al. (57) reported that pretreatment with antioxidant peptides could protect the hepatocytes from H_2_O_2_-induced cell death. It was achieved by reducing the ROS and MDA levels and enhancing the defense mechanism of endogenous antioxidant enzymes (SOD, CAT, and GSH-Px). Therefore, HBP extract demonstrates an excellent protective effect against oxidative damage during the differentiation of 3T3-L1 adipocytes.

## Conclusion

The study concludes that HBP extract could inhibit obesity-related enzymes, adipocyte differentiation, and adipogenesis in 3T3-L1cells. Enzymatic inhibition assay indicated that HBP extract exhibited good α-amylase, α-GLU, and lipase inhibition activities. Especially, the sample group showed even better enzyme inhibitory activity than the positive control drug. HBP extract inhibited the differentiation of 3T3-L1 preadipocytes by inducing cell cycle arrest in G0/G1 phase in our study. Furthermore, the results of oil red O staining showed that HBP extract reduced lipid accumulation in mature adipocytes. The anti-adipogenic potential of HBP extract is also illustrated in the attenuation of intracellular oxidative stress. Moreover, our results suggested that HBP extract could regulate the expression of mRNA and protein of adipogenic genes to have a lipid-lowering effect. The results revealed the anti-adipogenic potential of HBP extract and suggested that HBP extract could help to prevent obesity. Further studies are warranted to elucidate the potential mechanisms of action of the HBP extract *in vivo*.

## Data availability statement

The original contributions presented in the study are included in the article/Supplementary material, further inquiries can be directed to the corresponding author.

## Author contributions

YL, XD, and BC contributed to the conception and design of the study. BC, XD, and QL validation and investigation. XZ and HL performed the statistical analysis. XD wrote the first draft of the manuscript. BC wrote sections of the manuscript. YL resources, writing—review, editing, supervision, and funding acquisition. All authors contributed to manuscript revision, read, and approved the submitted version.

## Funding

This study was supported by the National Natural Science Foundation of China (No. 31560428, No. 31360378).

## Conflict of interest

The authors declare that the research was conducted in the absence of any commercial or financial relationships that could be construed as a potential conflict of interest.

## Publisher's note

All claims expressed in this article are solely those of the authors and do not necessarily represent those of their affiliated organizations, or those of the publisher, the editors and the reviewers. Any product that may be evaluated in this article, or claim that may be made by its manufacturer, is not guaranteed or endorsed by the publisher.
