# Supplementary figures and images for: Hulless barley polyphenol extract inhibits adipogenesis in 3T3-L1 cells and obesity related-enzymes
Source: Front Nutr. 2022 Aug 4;9:933068. doi: 10.3389/fnut.2022.933068 (PMC9389463; doi:10.3389/fnut.2022.933068)

**PPARγ**

**
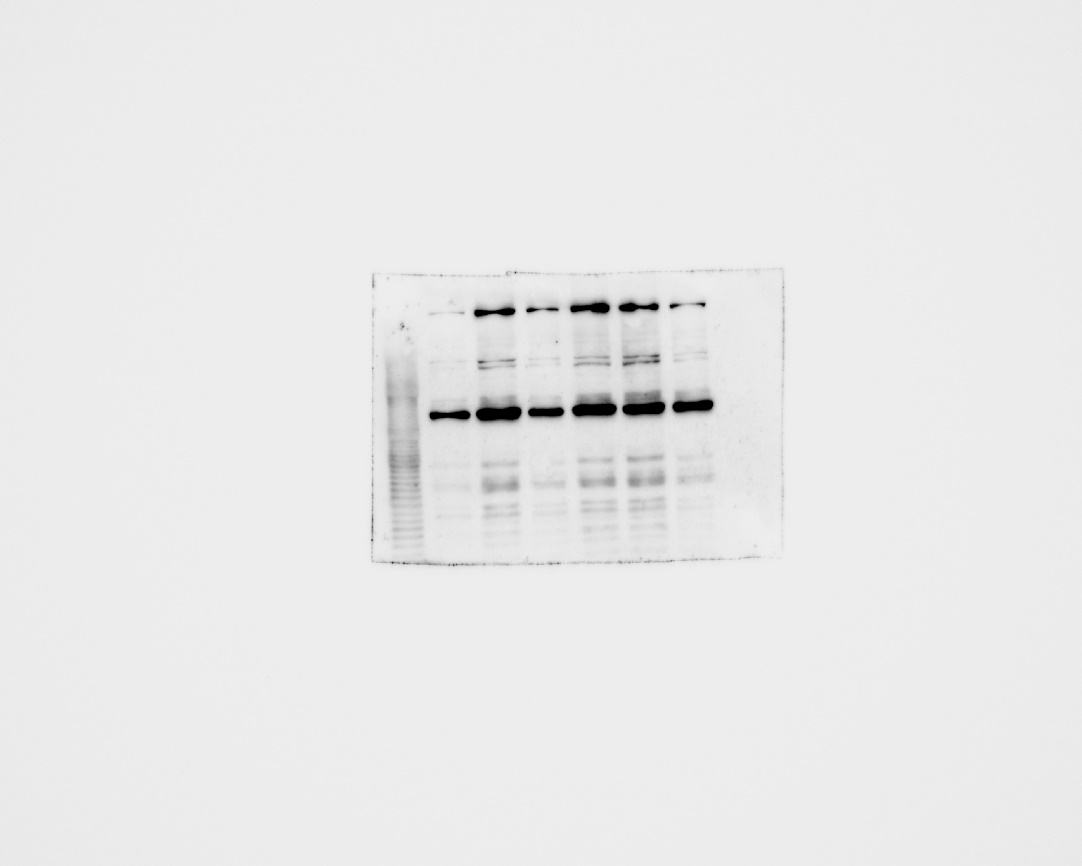
**

**C/EBPα**

**
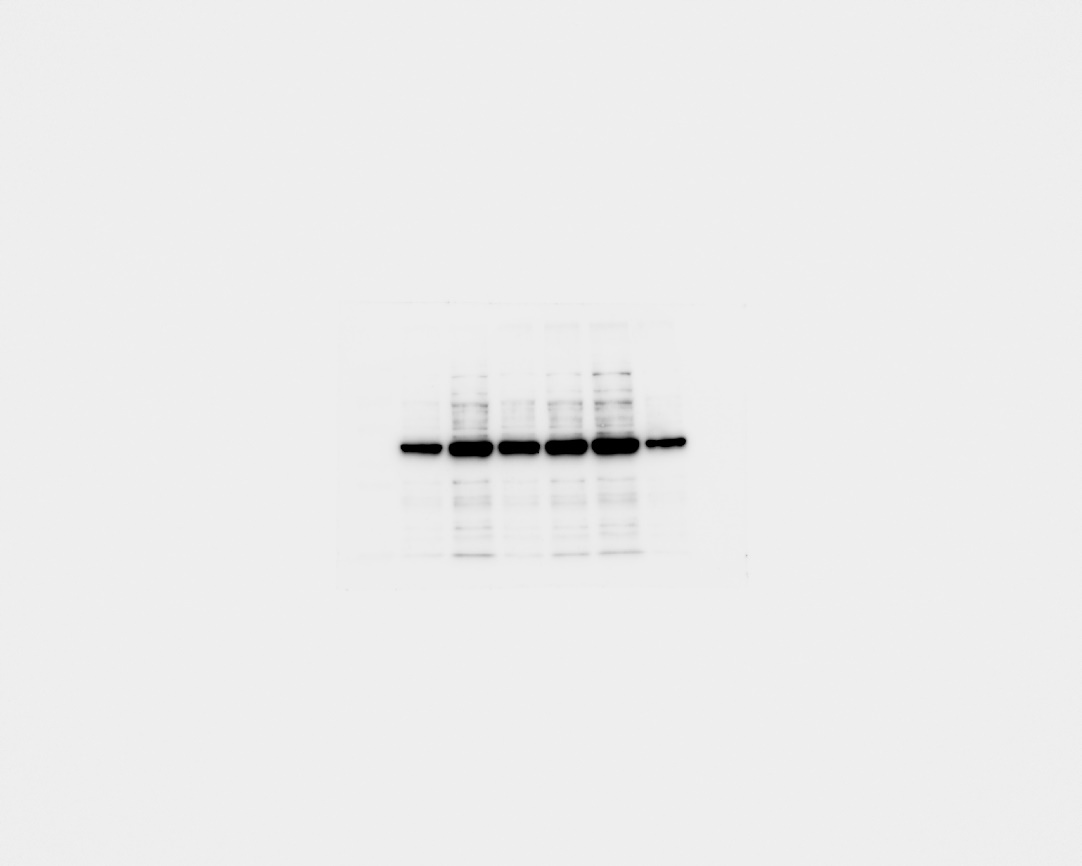
**

**FAS**

**
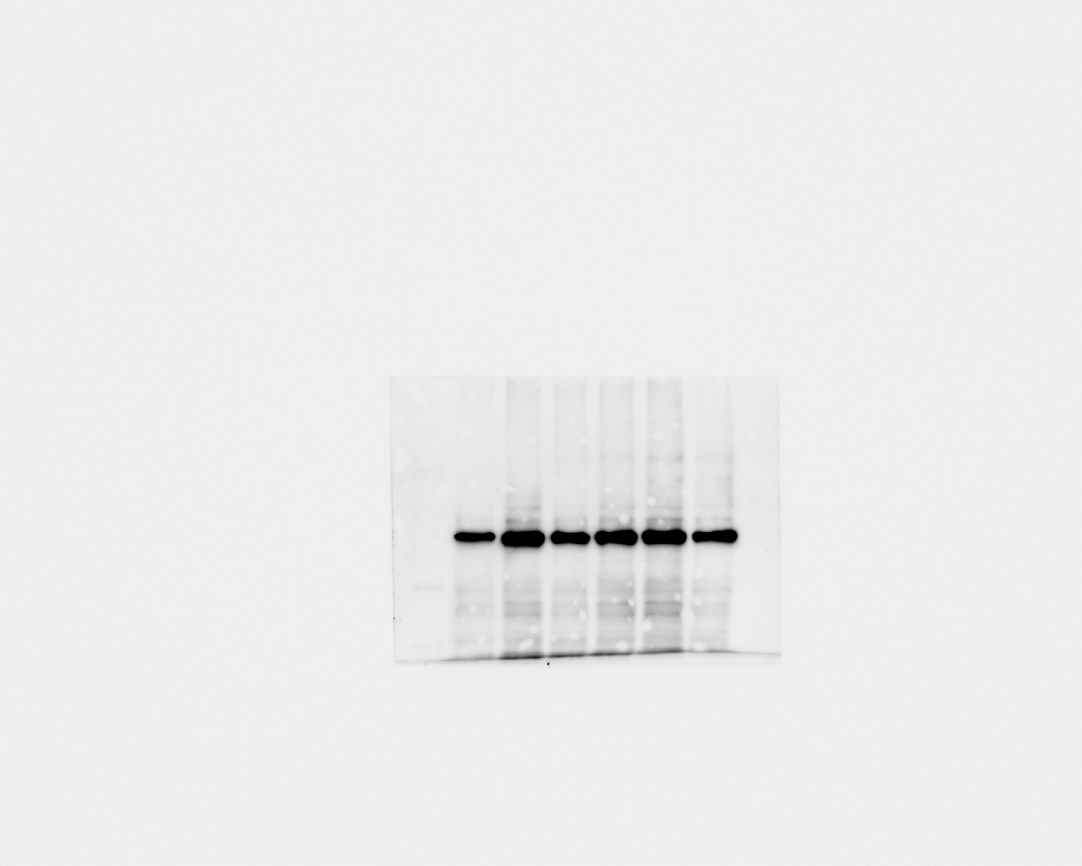
**

**ATGL**

**
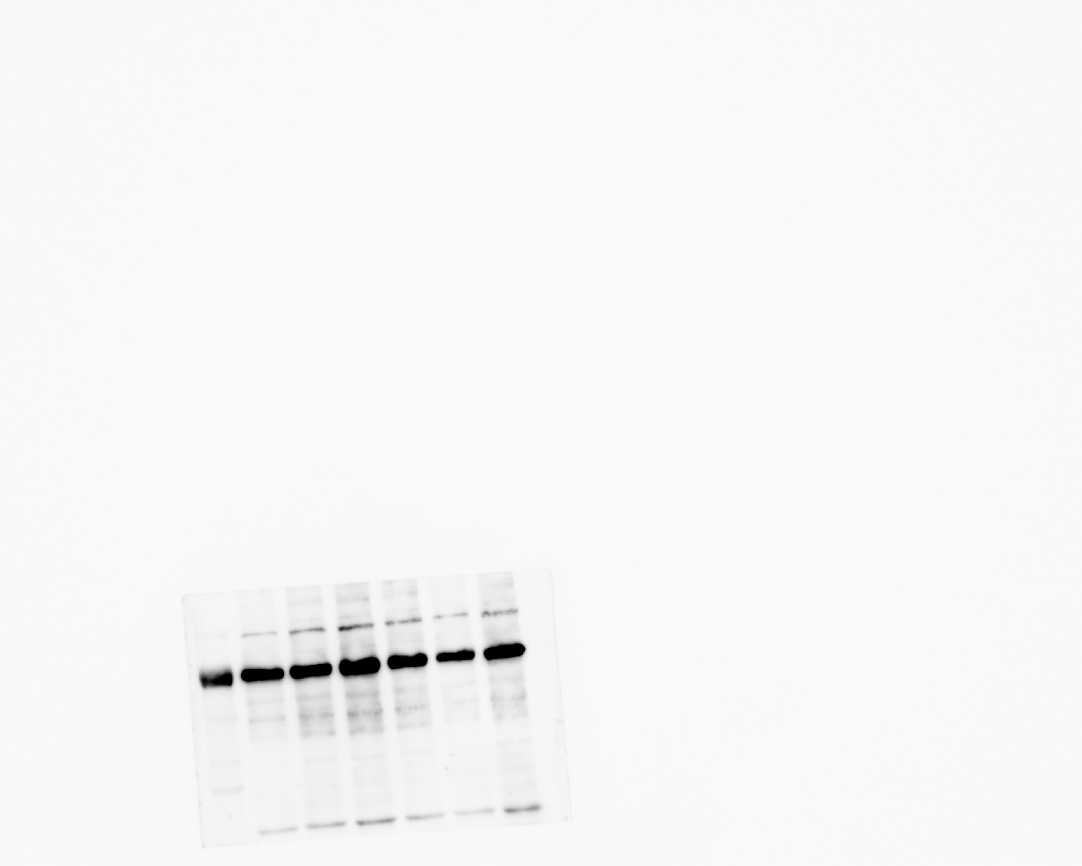
**

**FABP4**

**
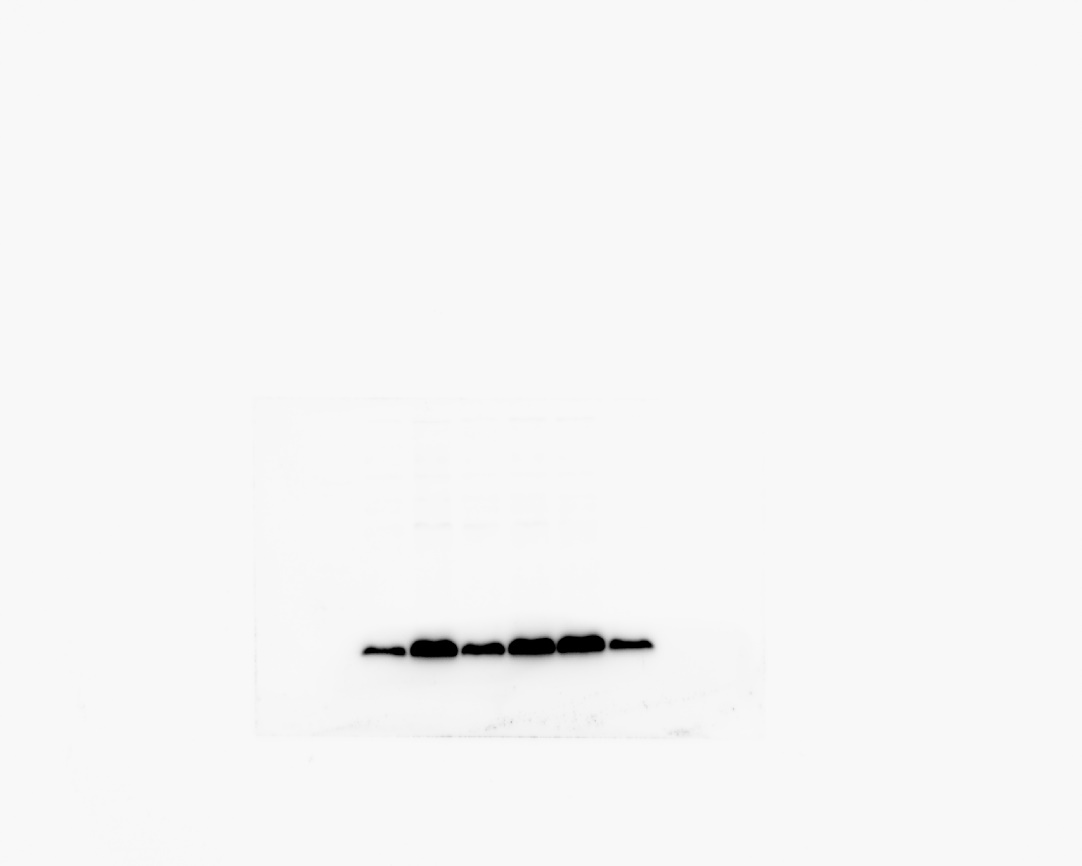
**

**β-actin**

**
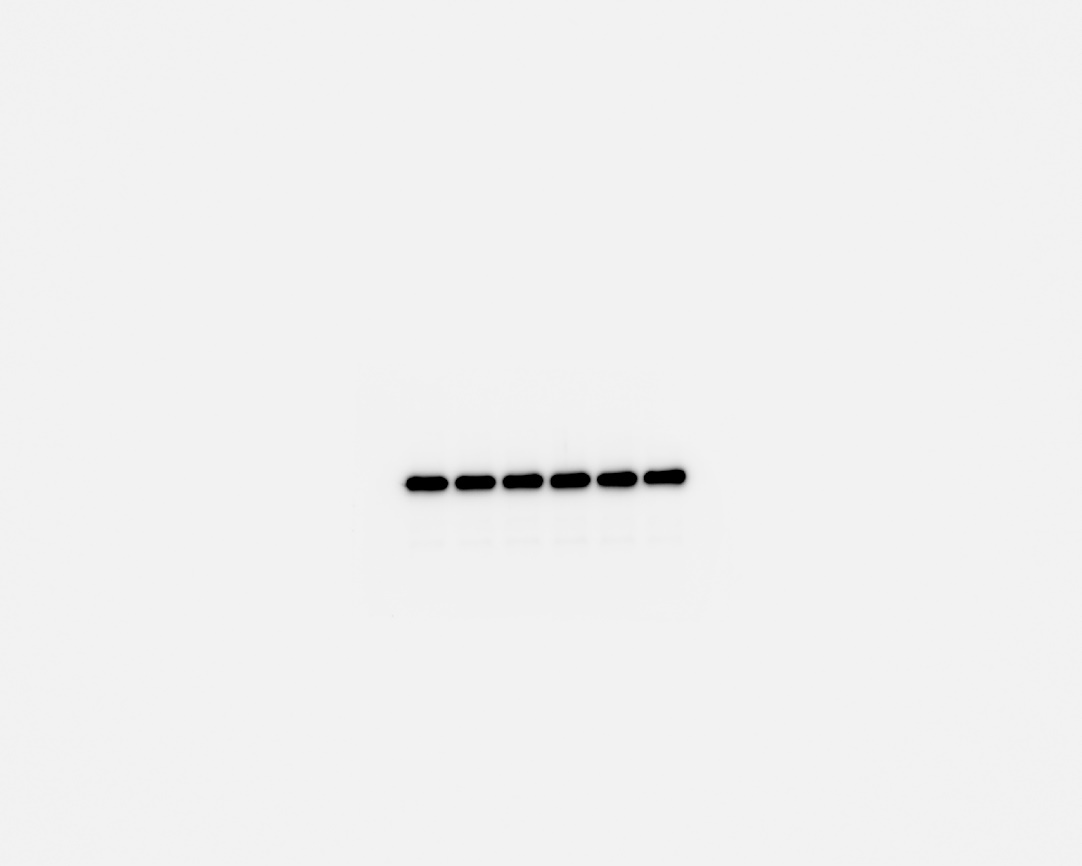
**

**Supplementary Figure 1.** Western blots primal pictures.

Supplement: Supplementary file 1 [file Table_1.DOCX]
