# Supplementary material for: Hulless barley polyphenol extract inhibits adipogenesis in 3T3-L1 cells and obesity related-enzymes
Source: Front Nutr. 2022 Aug 4;9:933068. doi: 10.3389/fnut.2022.933068 (PMC9389463; doi:10.3389/fnut.2022.933068)
Supplement: Supplementary file 3 [file Data_Sheet_1.PDF]

List of figures

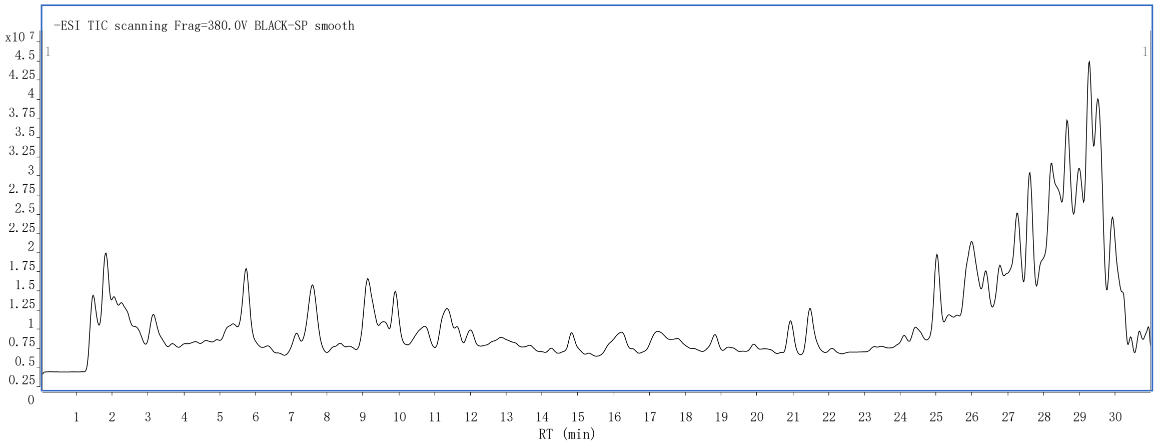

Supplementary Figure 1 Total ion chromatogram of hull-less barley polyphenol (HBP) extract.

1. 6''-O-Caffeoylastragalin

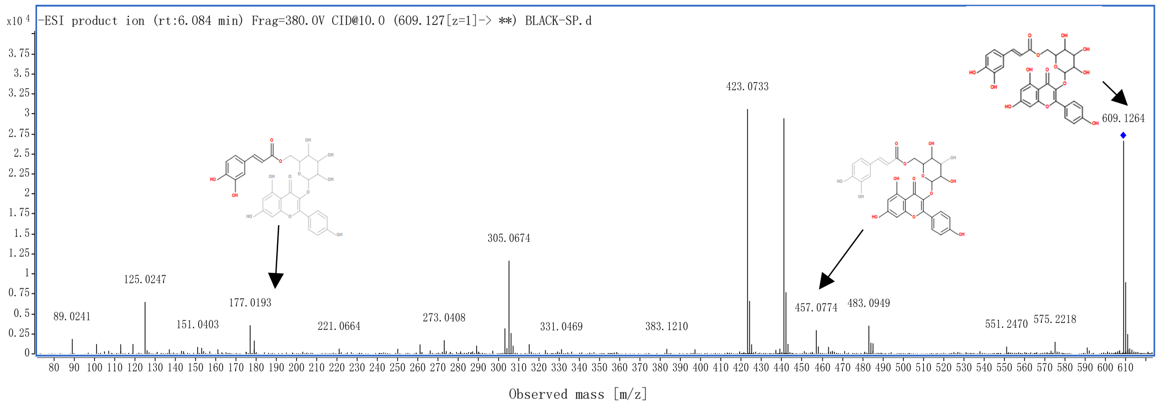

2. Vanillic acid (4-Hydroxy-3-methoxy-benzoic acid)

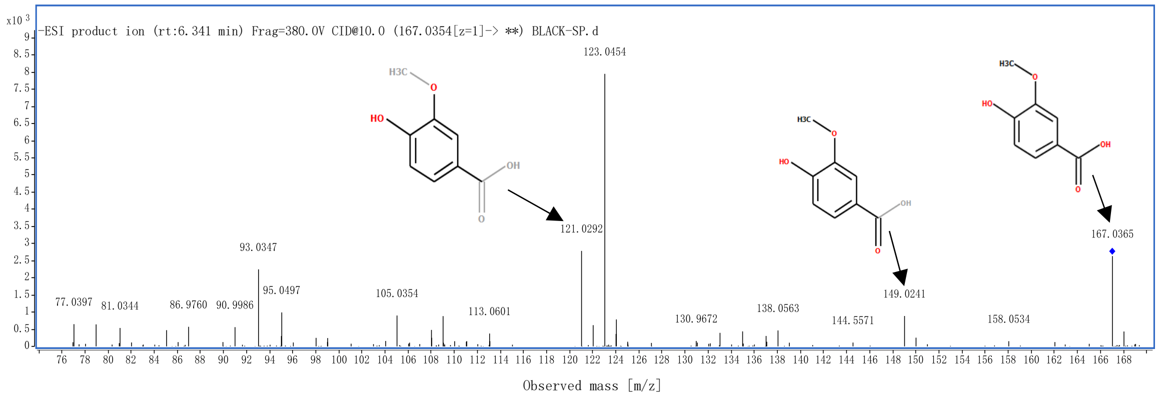

3. Homogentisic acid

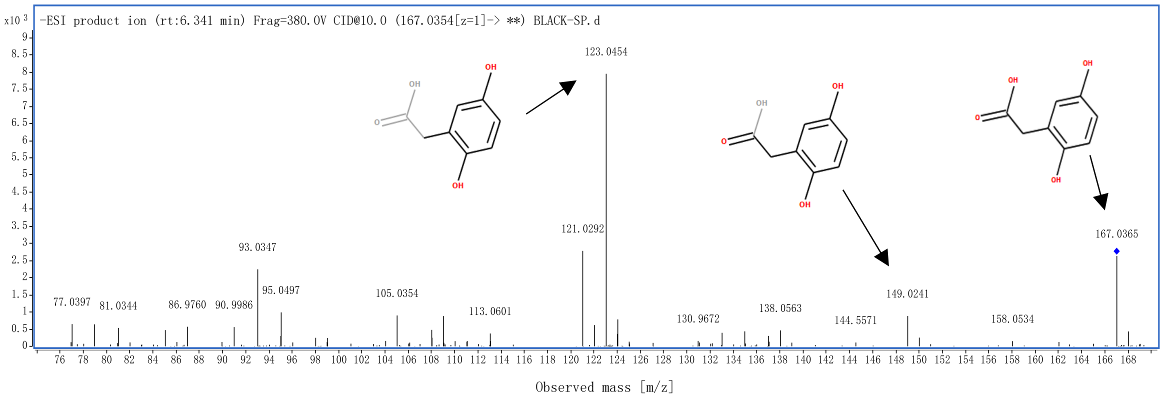

4. Protocatechuic acid

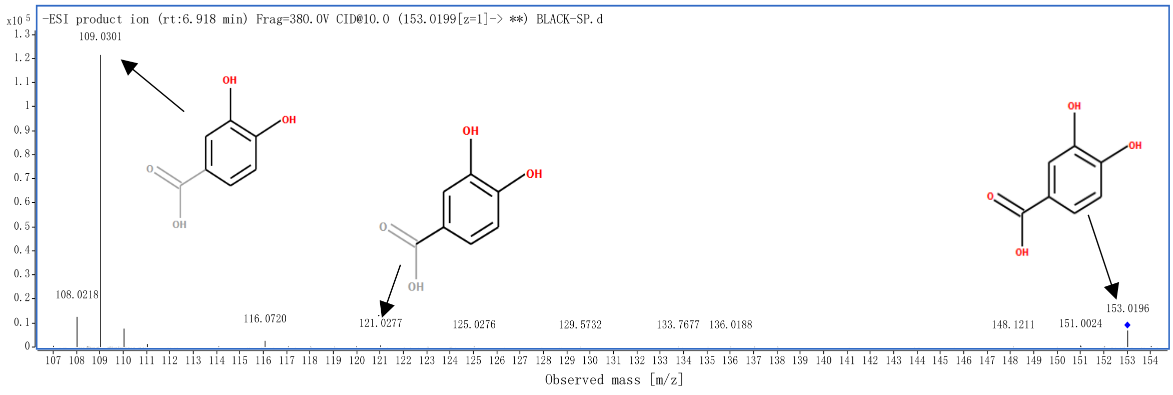

5. (-)-epigallocatechin

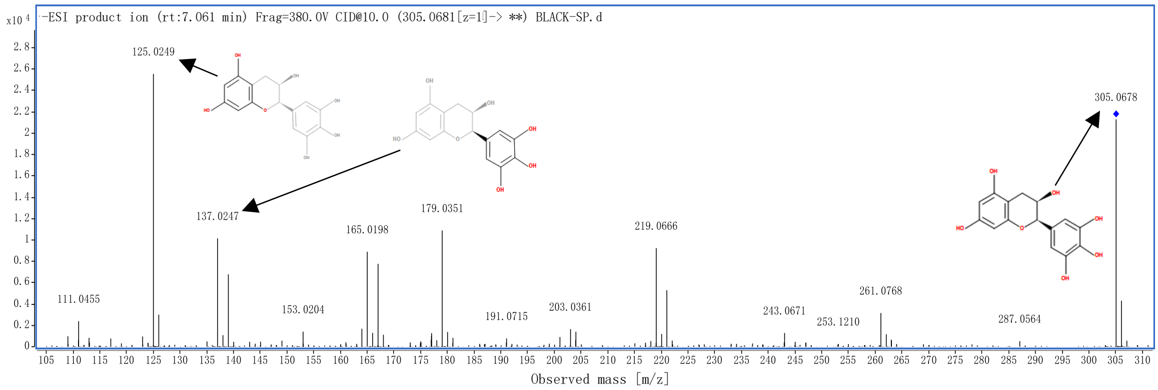

6. Leiocarposide

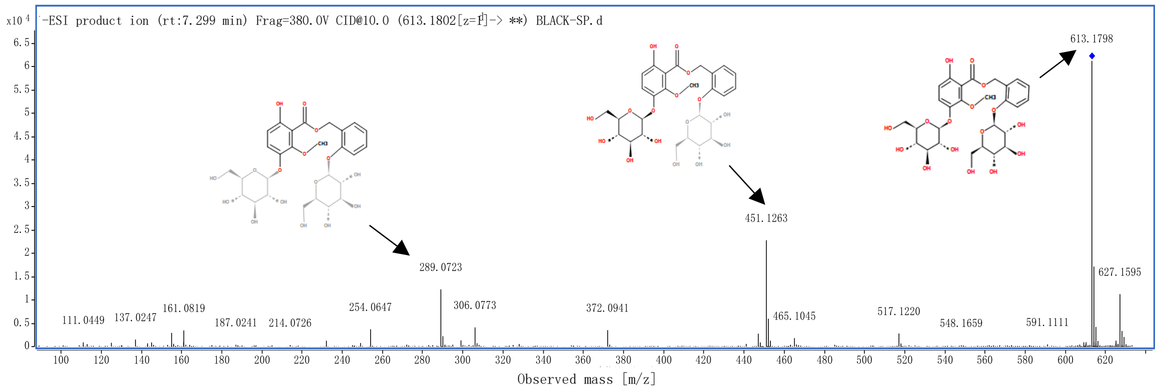

7. Tetracenomycin B2

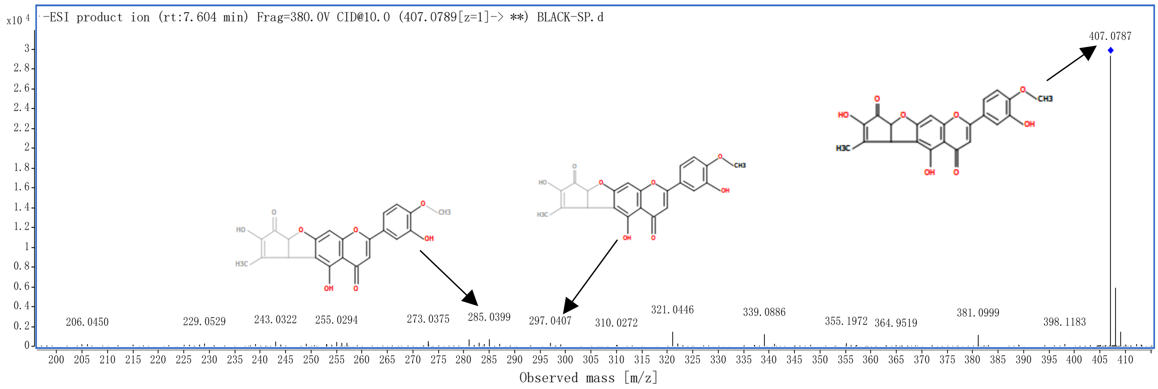

8. Tiliroside

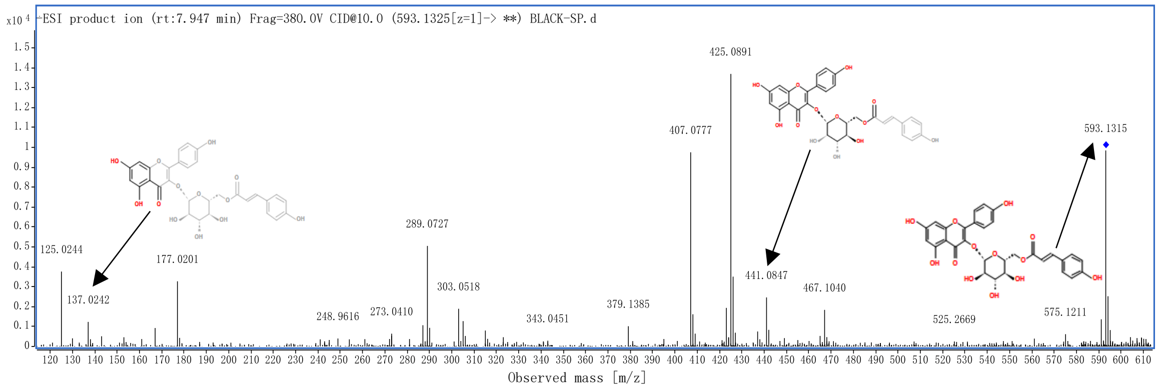

9. Gallocatechin-(4alpha->8)-catechin-(4alpha->8)-catechin

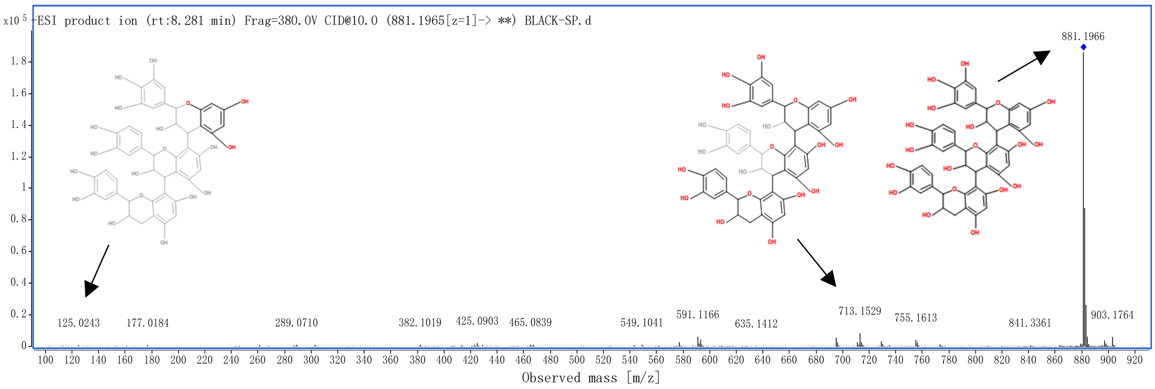

10. Sesamol

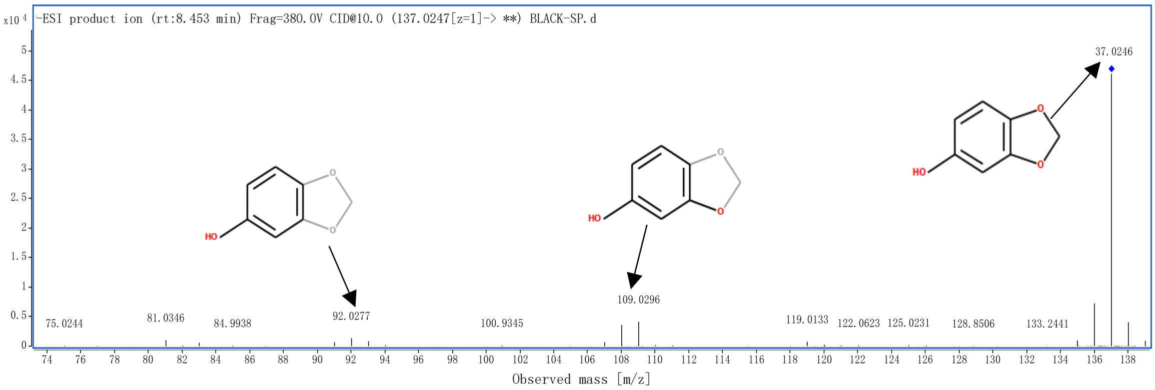

11. p-Hydroxybenzaldehyde

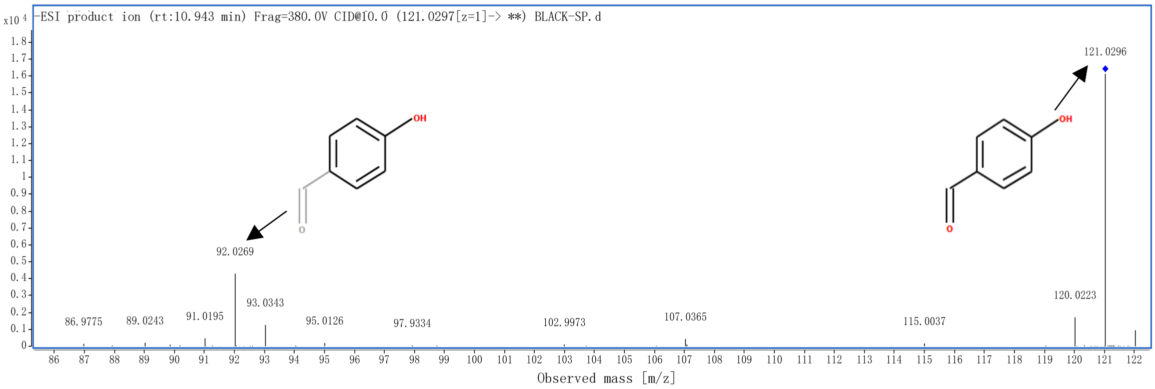

12. Procyanidin B2

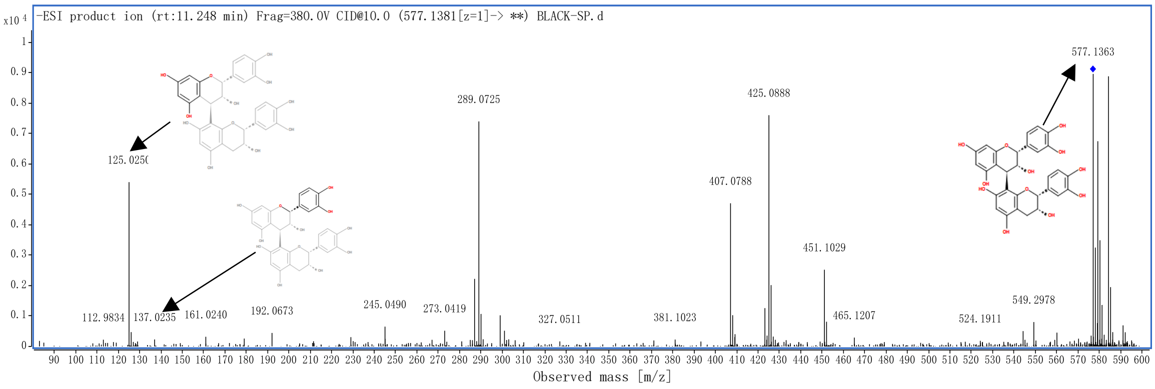

13. p-Coumaroylagmatine

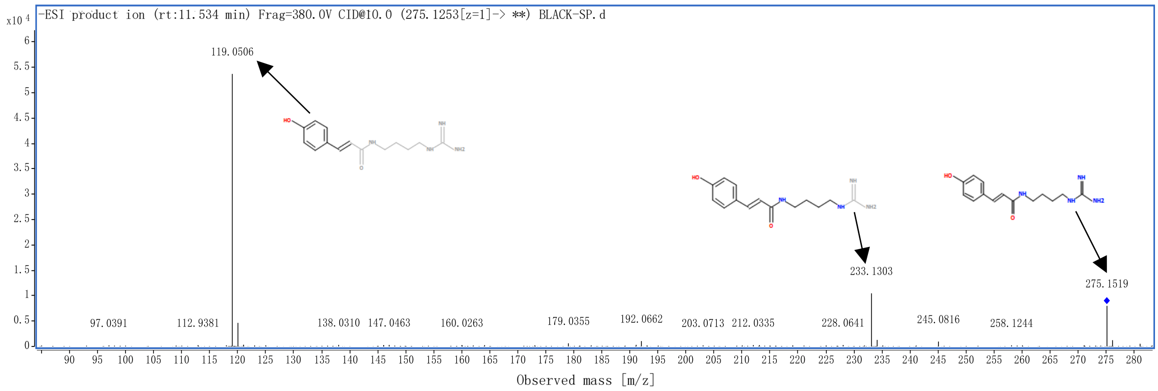

14. Neoeriocitrin

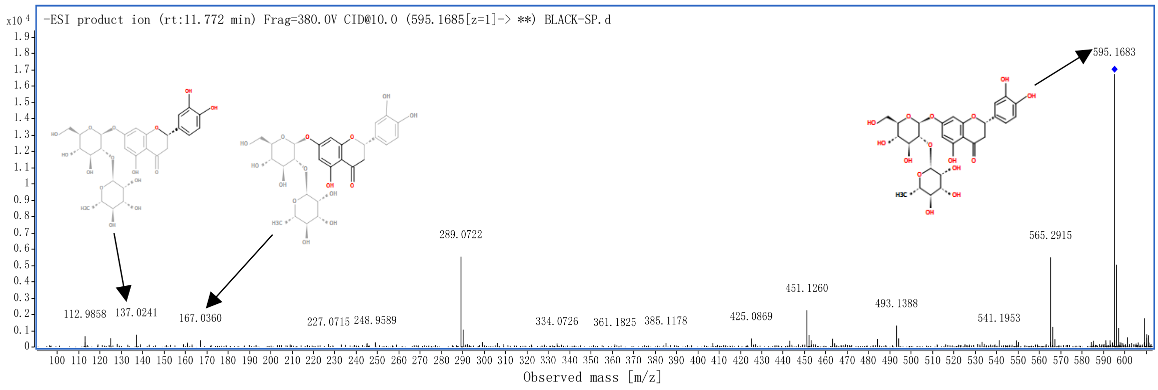

15. Epicatechin 3'-O-glucuronide

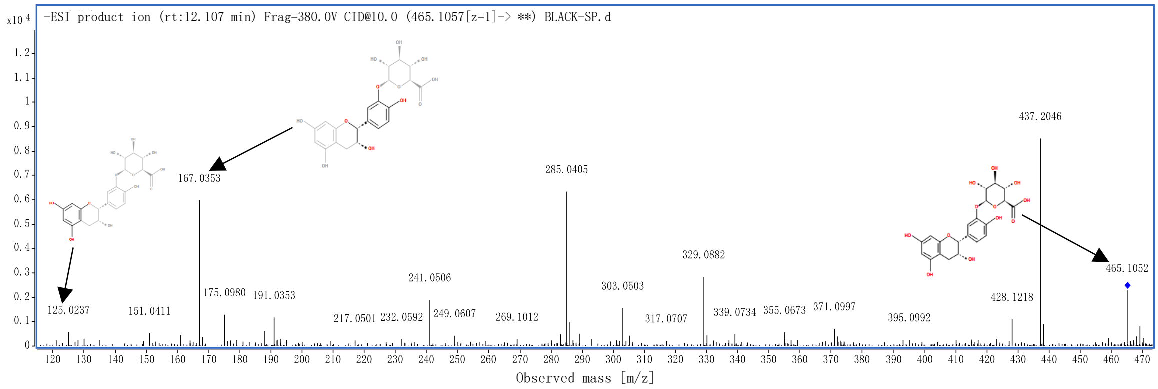

16. Plantagoside

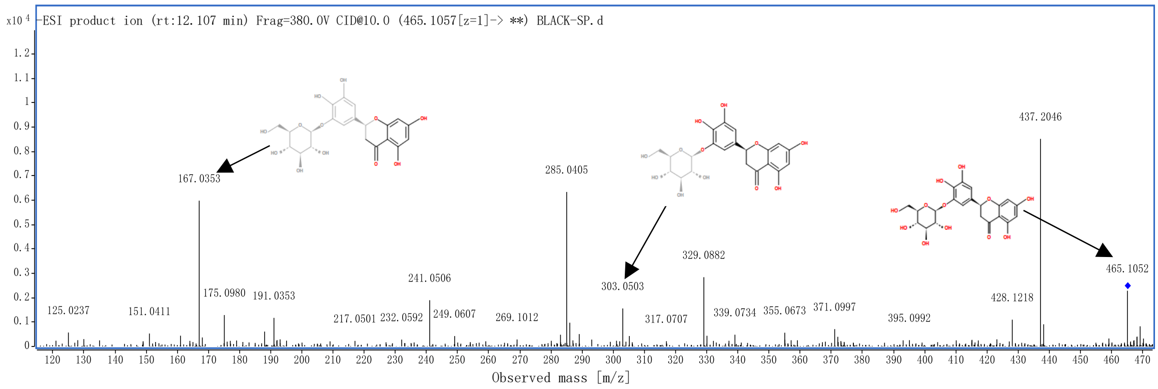

17. Glucodistylin

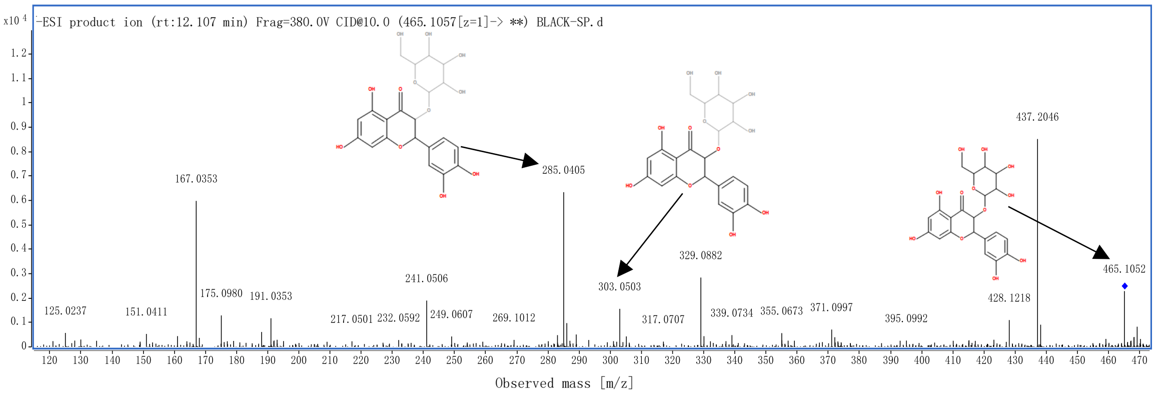

18. Dihydroisorhamnetin

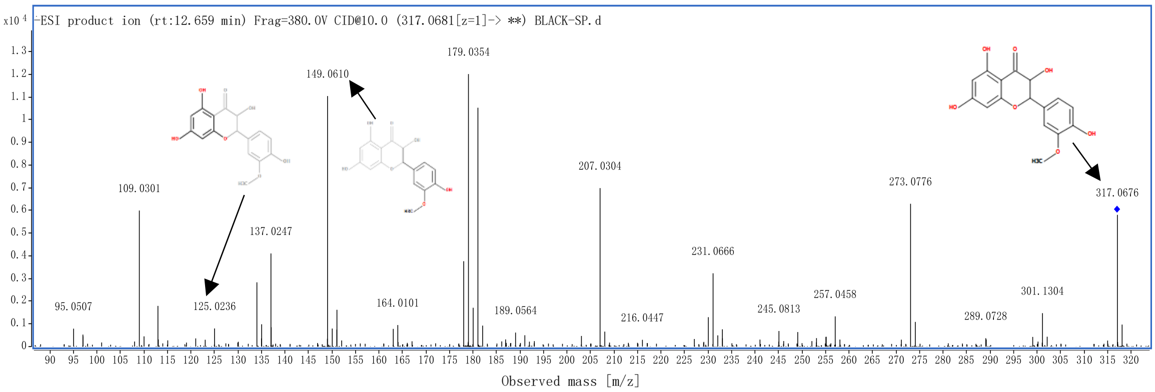

19. 4'-O-Methyl-(-)-epicatechin 3'-O-glucuronide

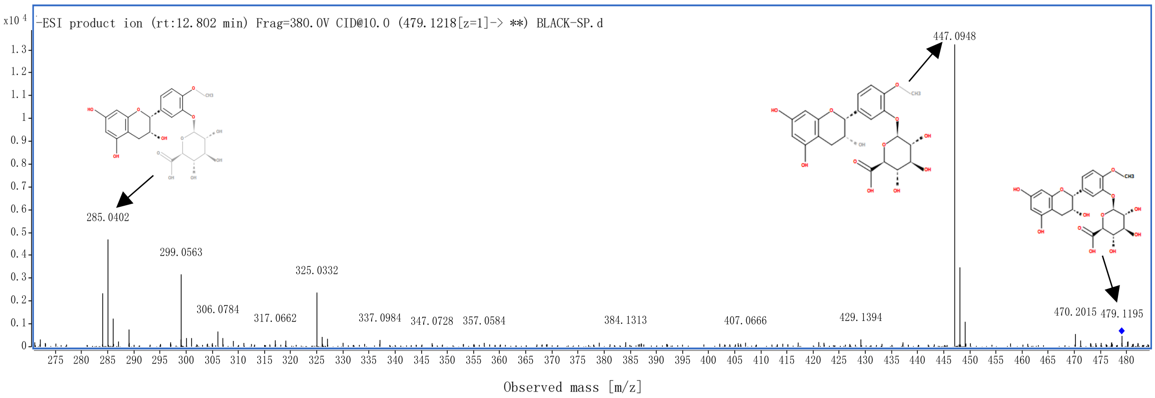

20. Naringenin 7-O-glucuronide

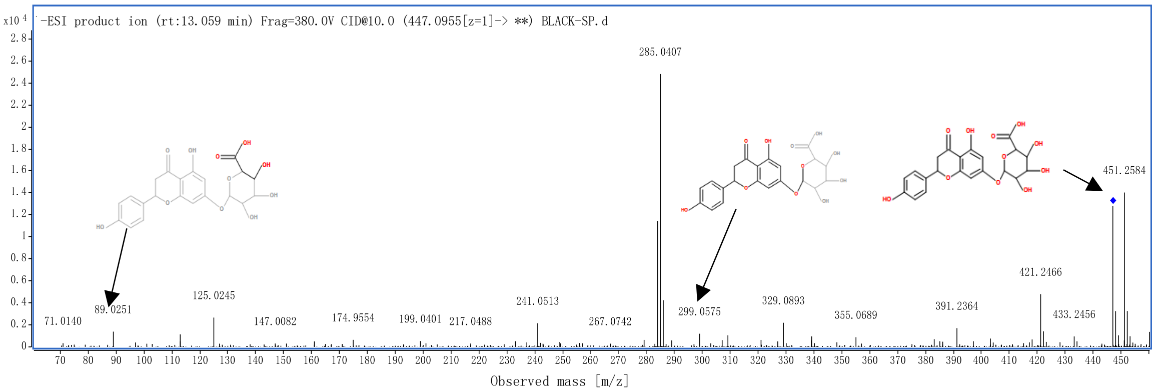

21. Astragalin

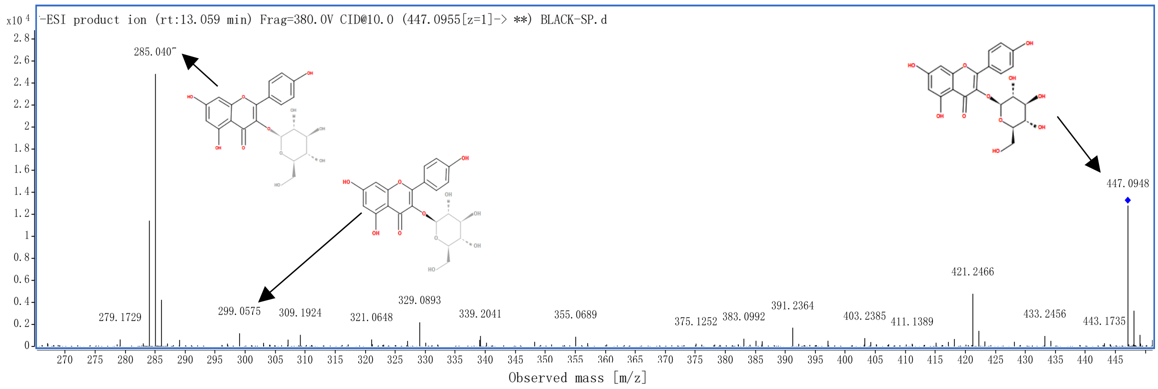

22. Vanillic acid

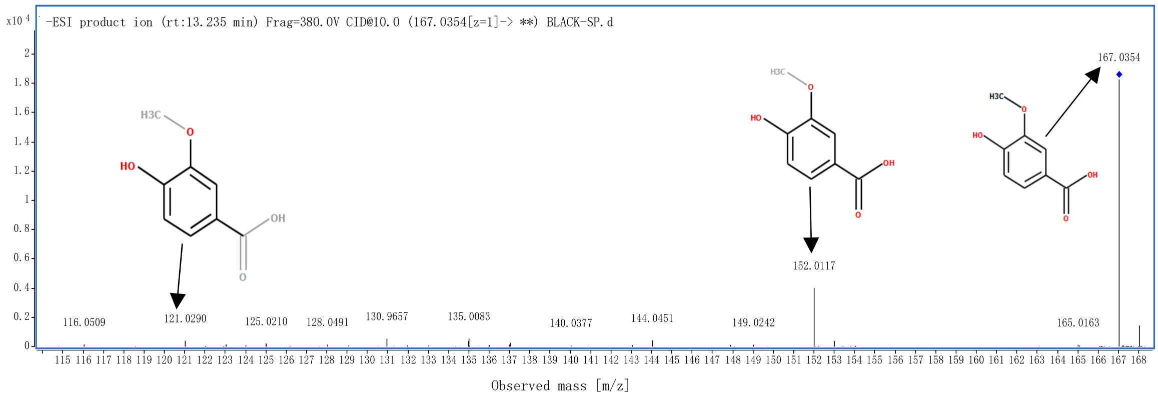

23. Methyl 3-(2,3-dihydroxy-3-methylbutyl)-4-hydroxybenzoate

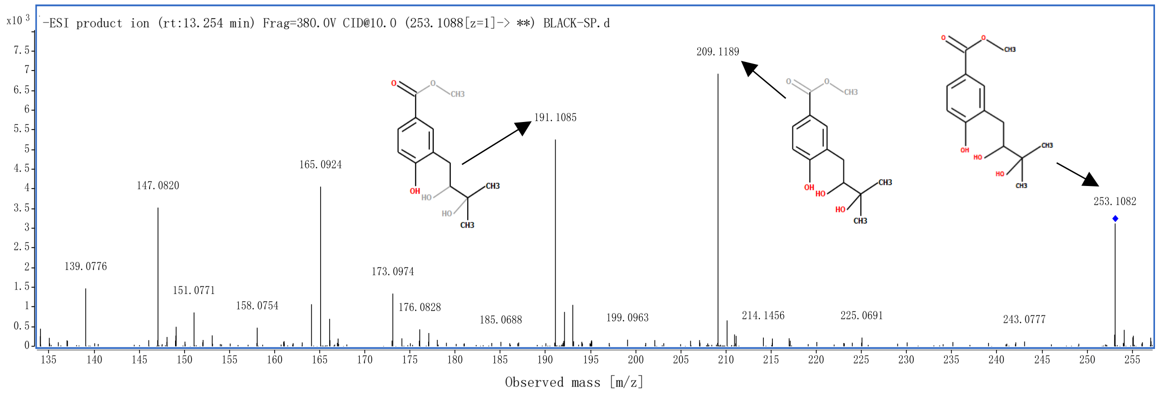

24. Cinnamtannin A1

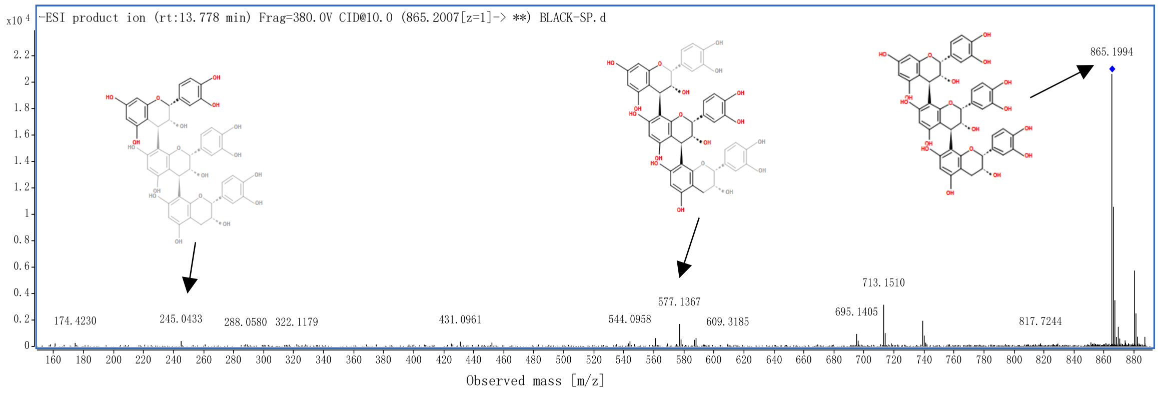

25. Kaempferol 3-(6-acetylgalactoside)

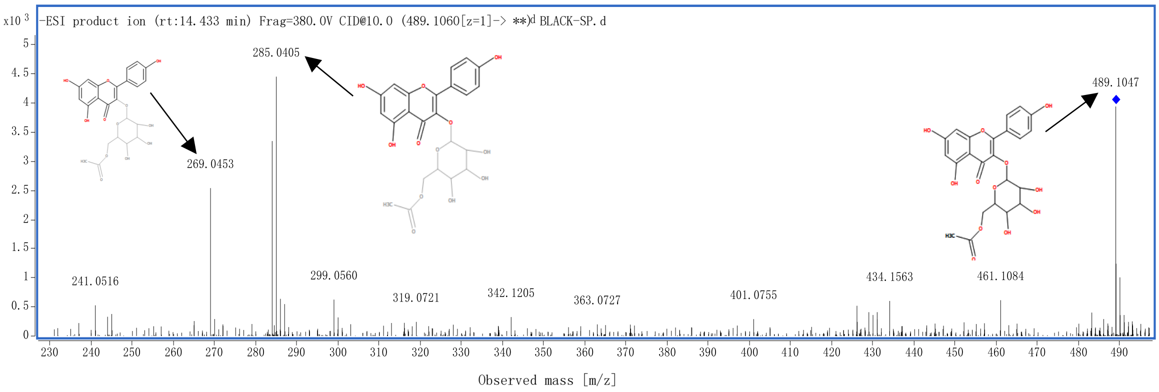

26. 4',8-Dimethylgossypetin 3-glucoside

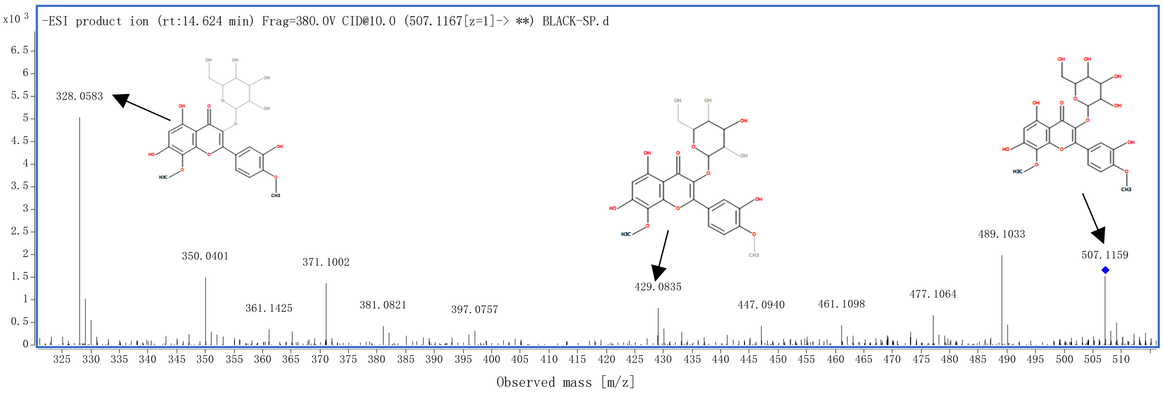

27. 6-Hydroxypelargonidin 3-rutinoside

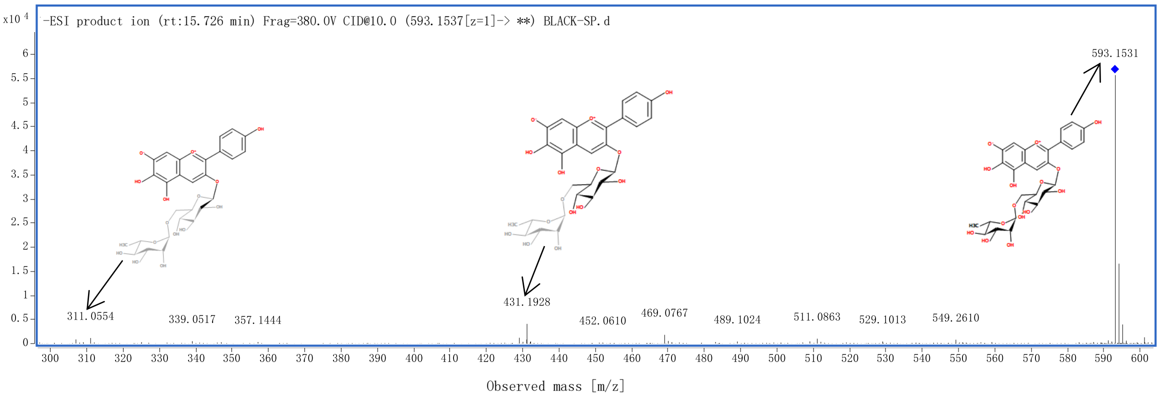

28. Tomentin 6-galactoside

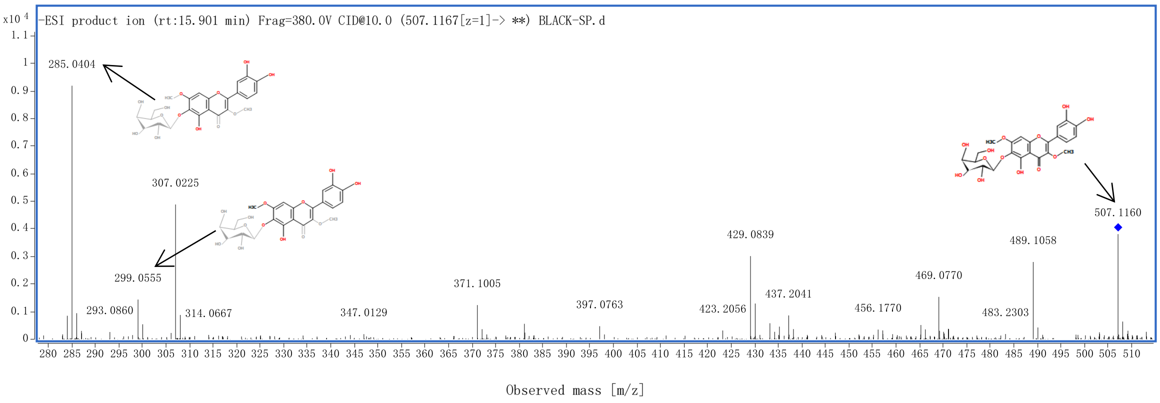

29. Quercetin 3-xylosyl-(1->2)-alpha-L-arabinofuranoside

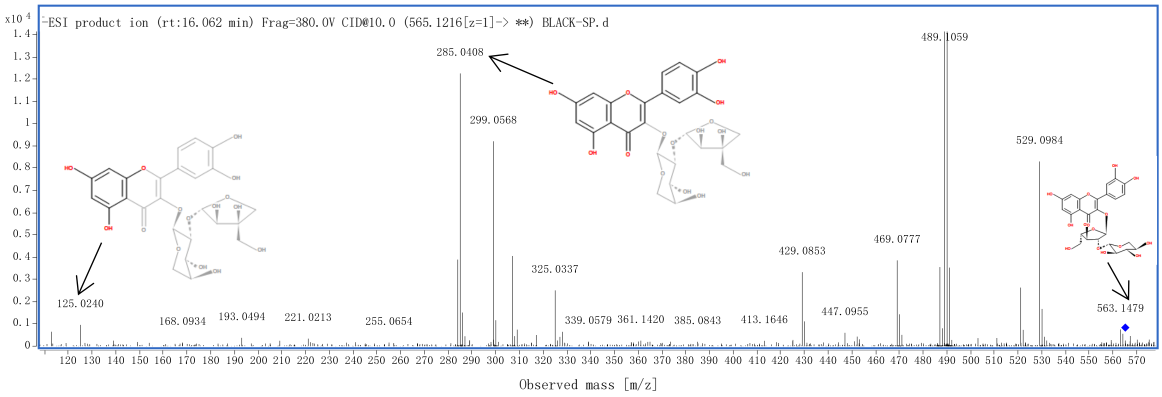

30. Leucodelphinidin 3-O-(beta-D-glucopyranosyl-(1->4)-alpha-L-rhamnopyranoside)

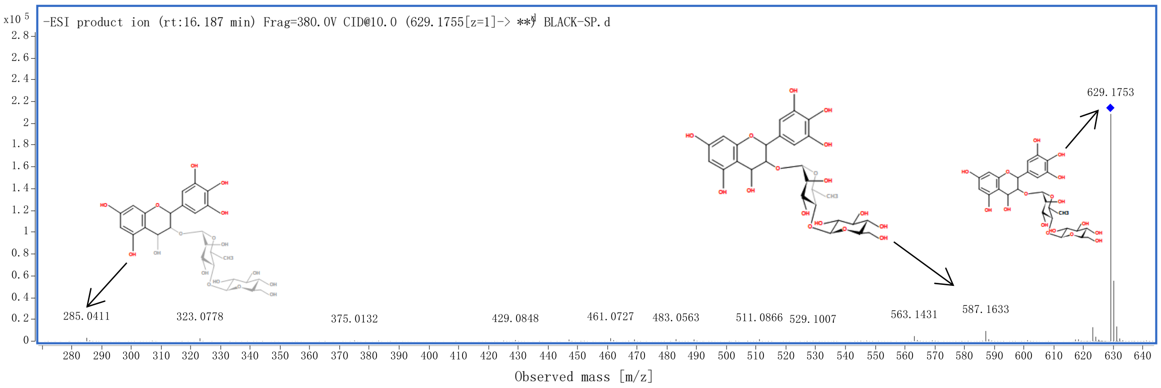

31. Ferulic acid (3-(4-Hydroxy-3-methoxyphenyl)-2-propenoic acid)

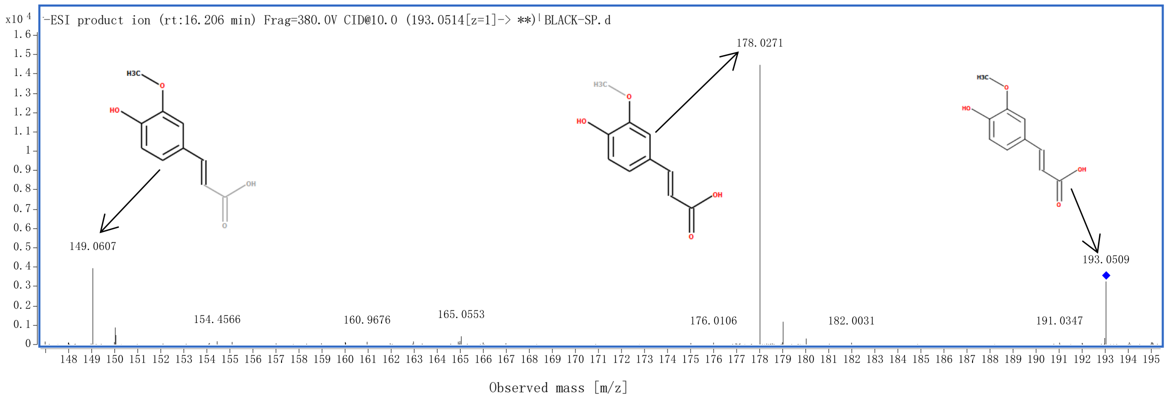

32. Isoferulic acid

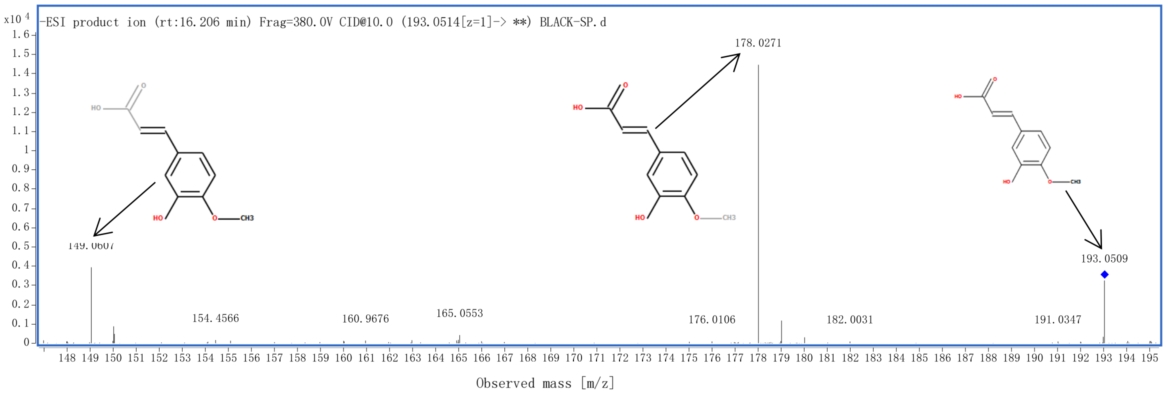

33. Kaempferol 3-(6-acetylgalactoside)

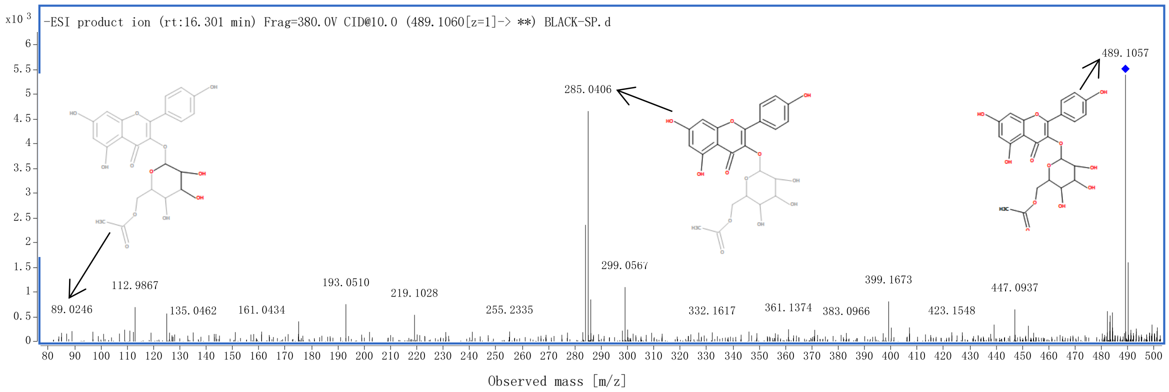

34. 3,5-di-O-(beta-Glucopyranosyl) pelargonidin 6''-O-4, 6'''-O-1-cyclic malate

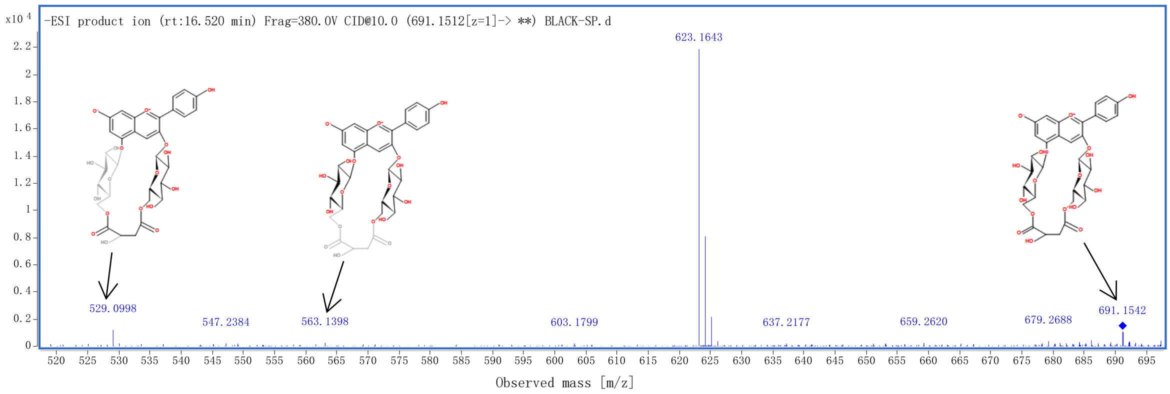

35. Typhaneoside

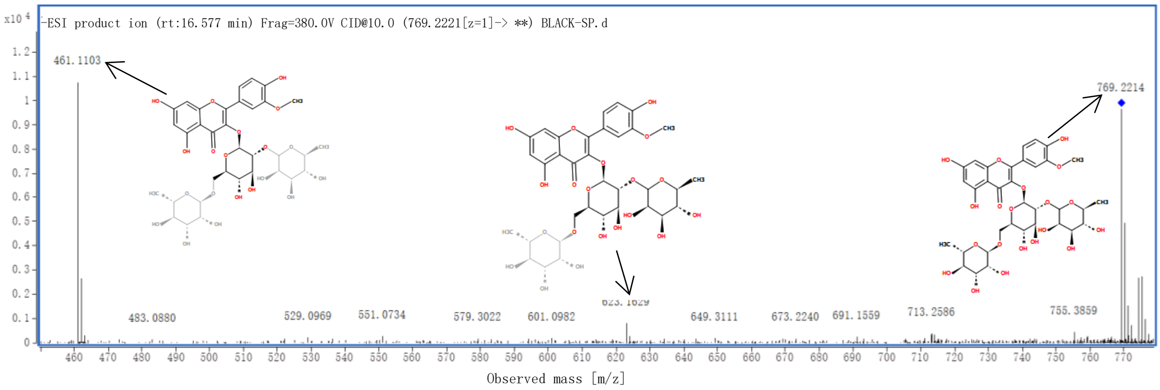

36. Isorhamnetin 3-rutinoside 4'-rhamnoside

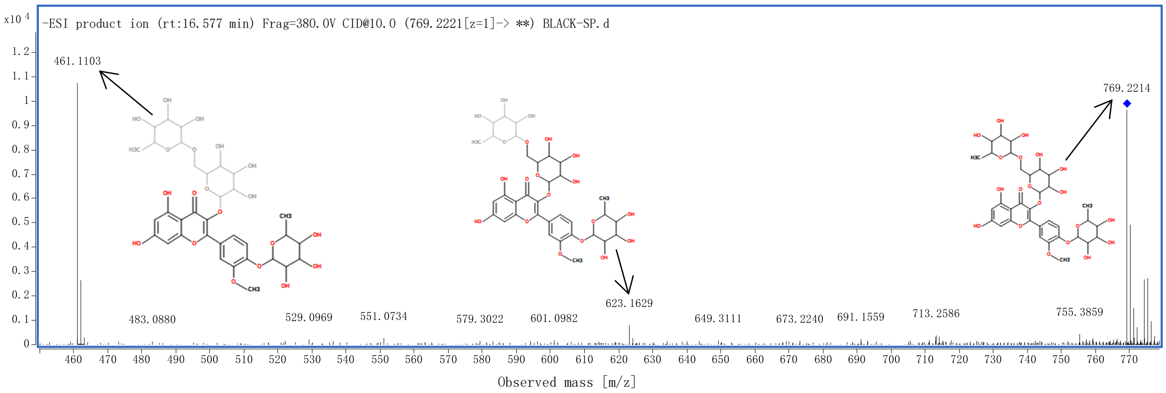

37. Quercetin (3 3' 4' 5 7-pentahydroxyflavone)

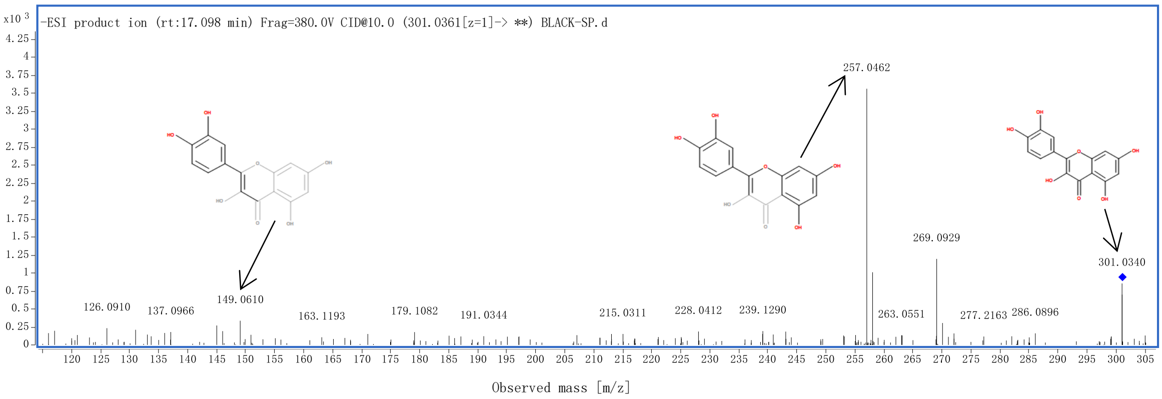

38. 6-Hydroxykaempferol

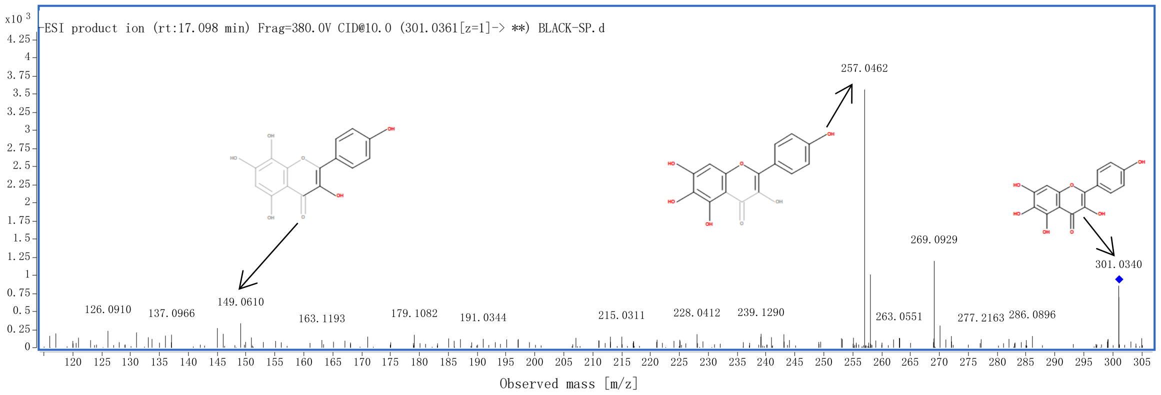

39. Glucosyl-vitexin

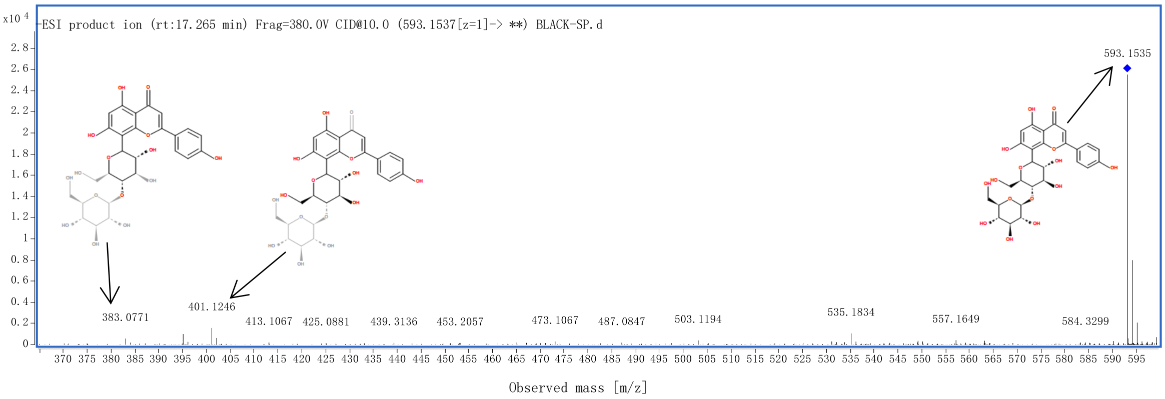

40. Kaempferol 3-rutinoside

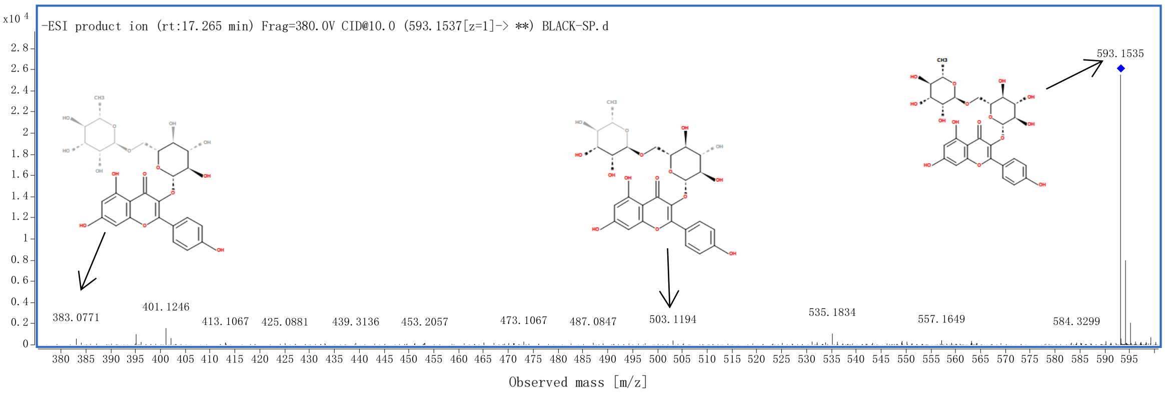

41. Graveobioside B

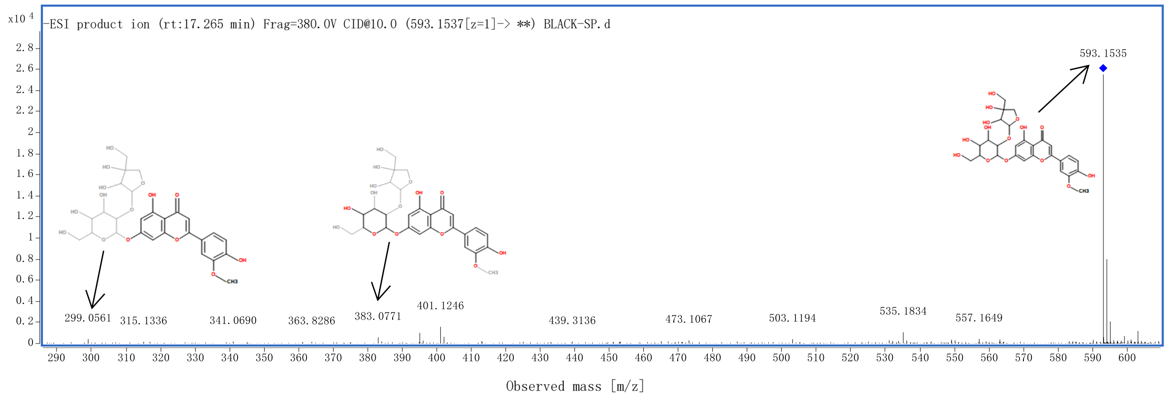

42. 5,3',4'-Trihydroxy-7-methoxy-4-phenylcoumarin 5-O-xylosyl-(1->6)-glucoside

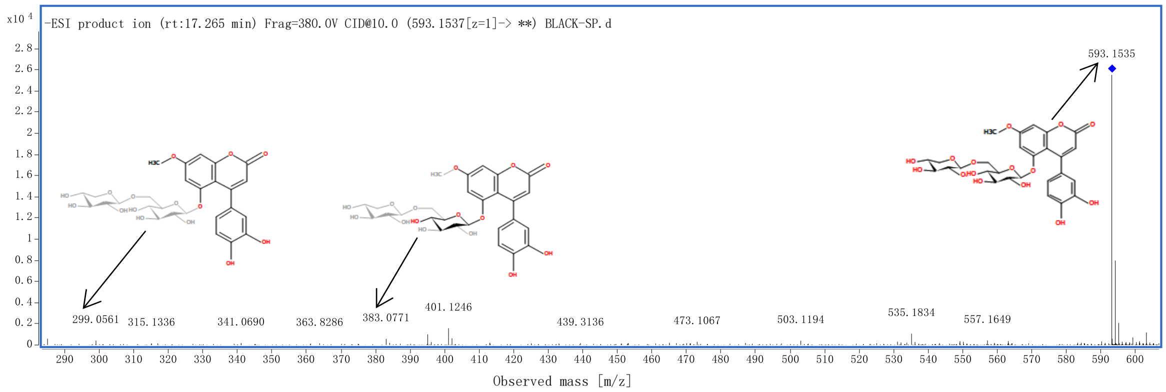

43. Limocitrin 3,7-diglucoside

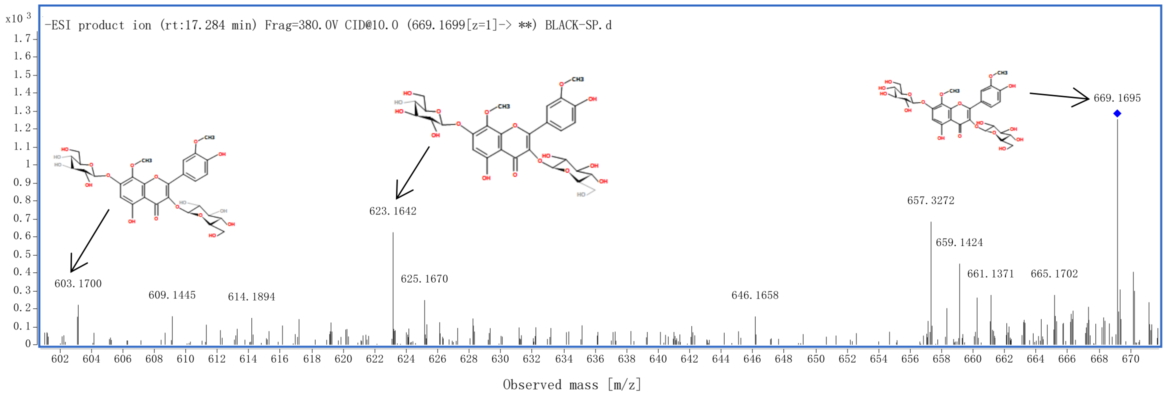

44. (7'R)-(+)-Lyoniresinol 9'-glucoside

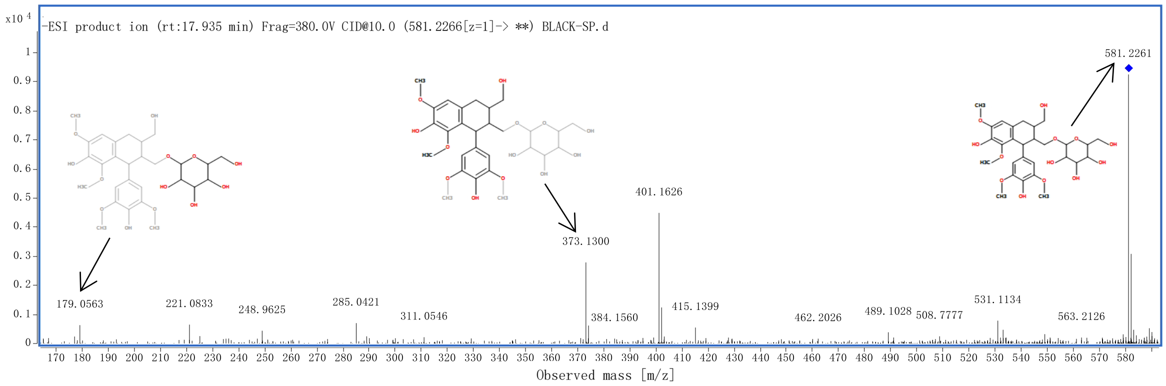

45. Narcissoside

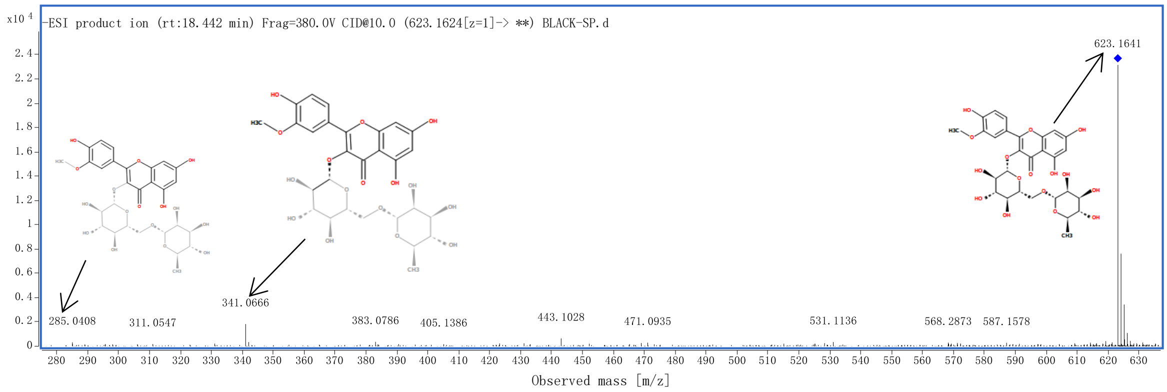

46. Pasternoside

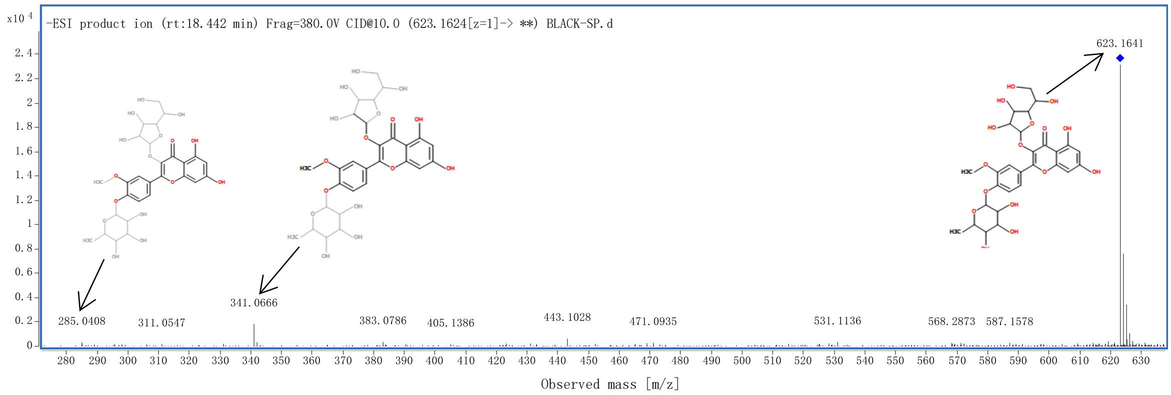

47. Kaempferol (3 4' 5 7-tetrahydroxyflavone)

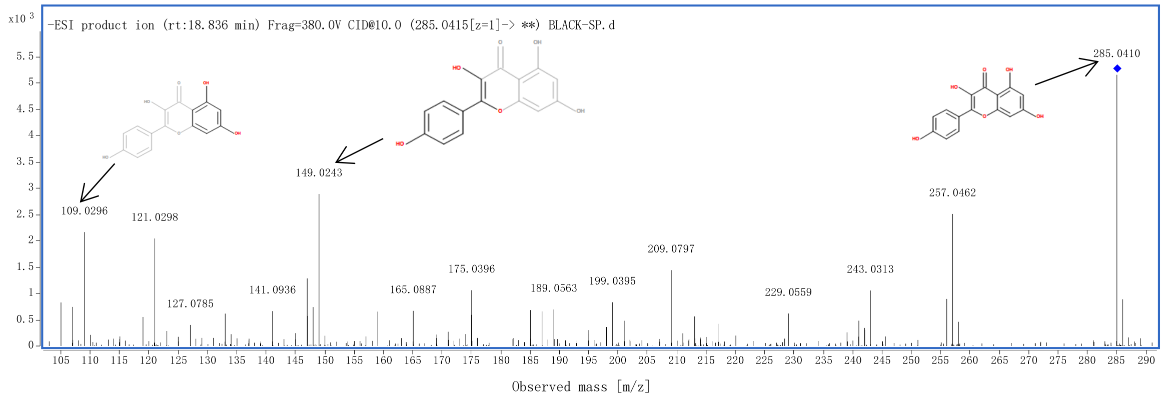

48. Fisetin

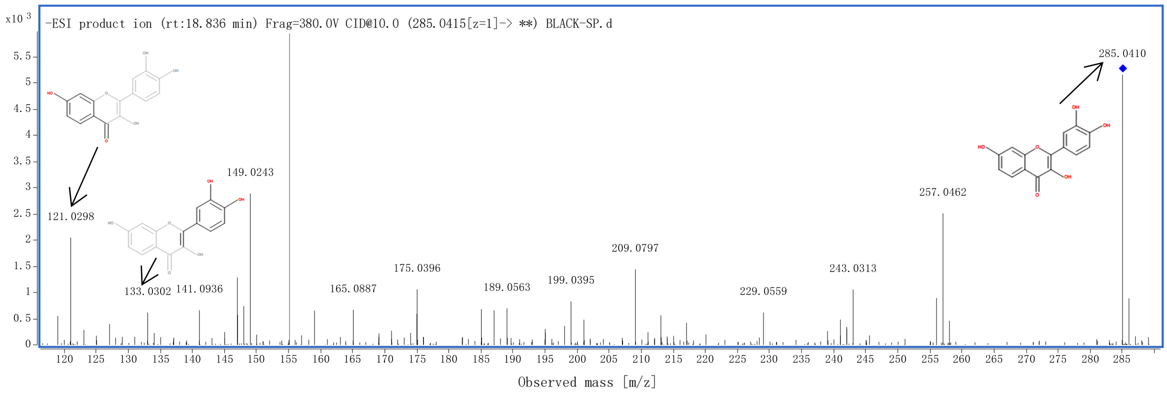

49. Cis-Mulberroside A

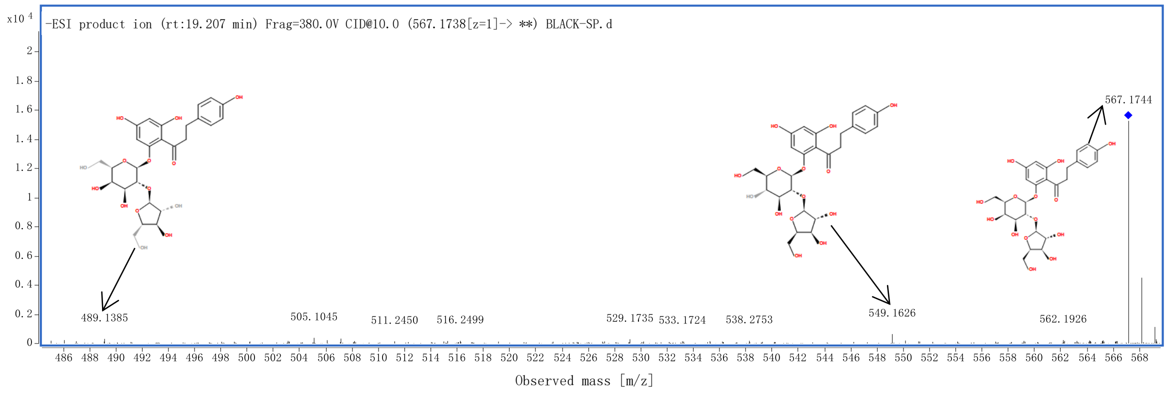

50. Glyphoside

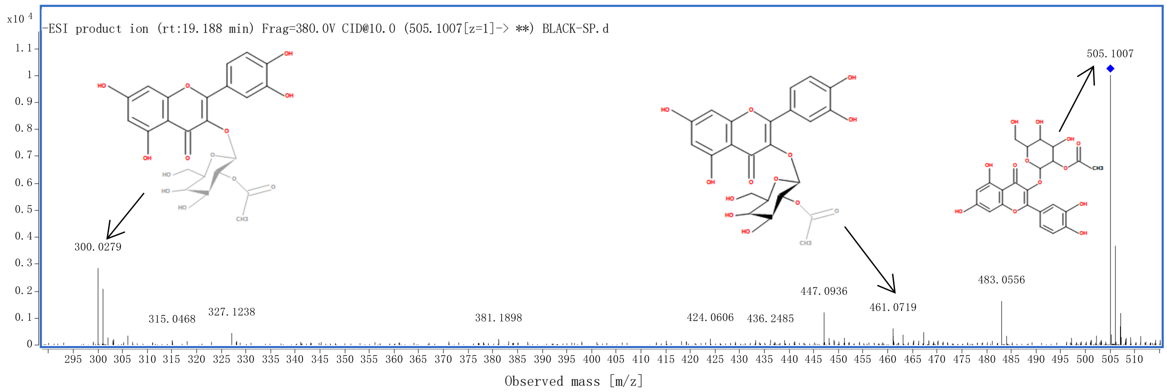

51. Tectoridin (Tectorigenin 7-glucoside；Chrysoeriol-O-hexoside)

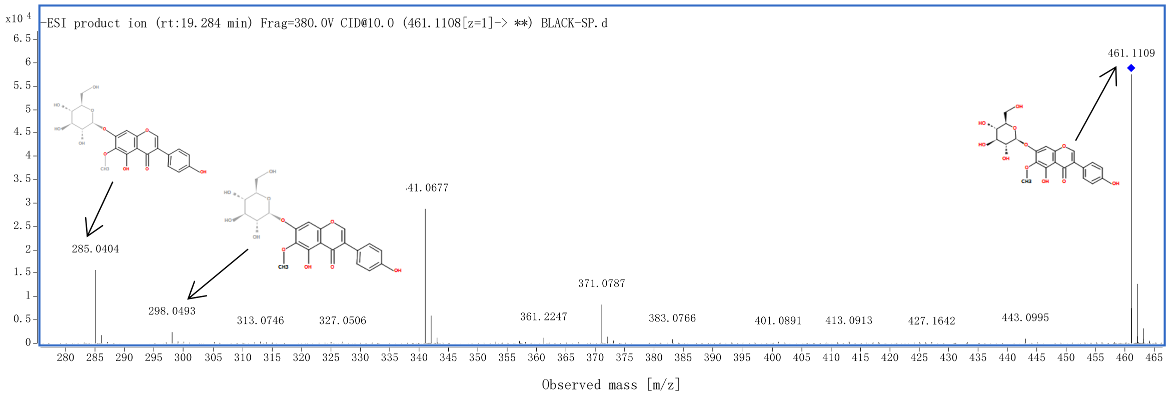

52. Clitorin

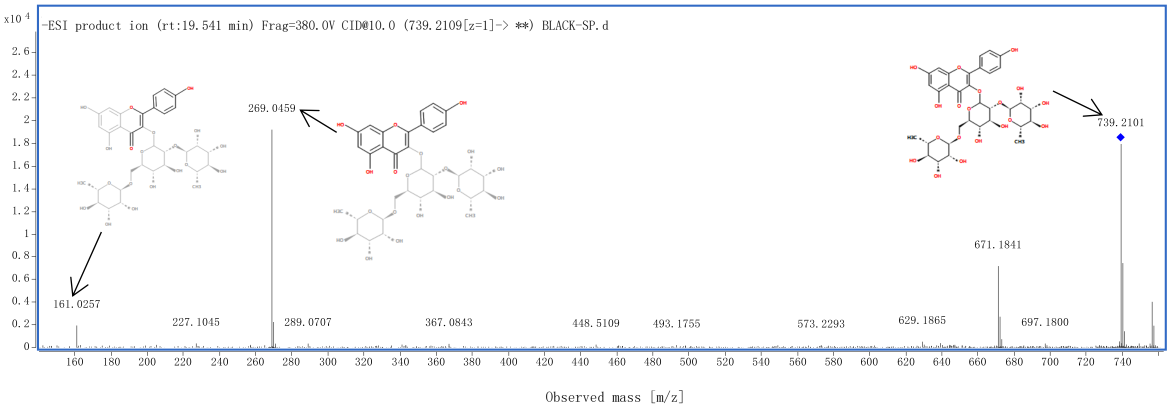

53. Kaempferol 3-(2''-rhamnosylrutinoside)

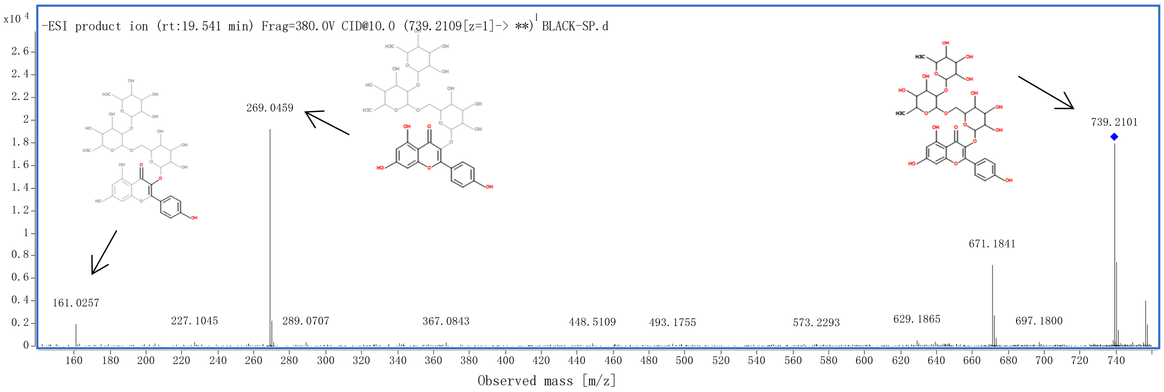

54. Isorhamnetin 3-O-[a-L-rhamnopyranosyl-(1->3)-a-L-rhamnopyranosyl-(1->6)-b-D-glucopyranoside]

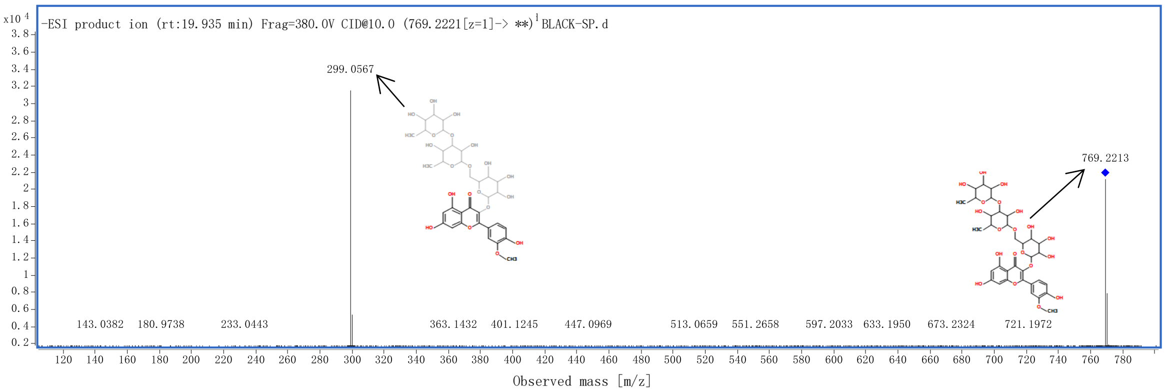

55. Kaempferitrin

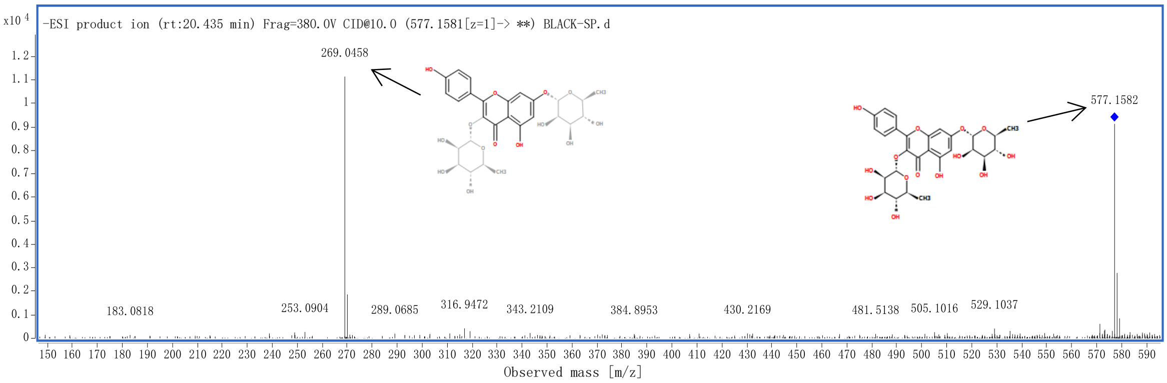

56. Rhoifolin

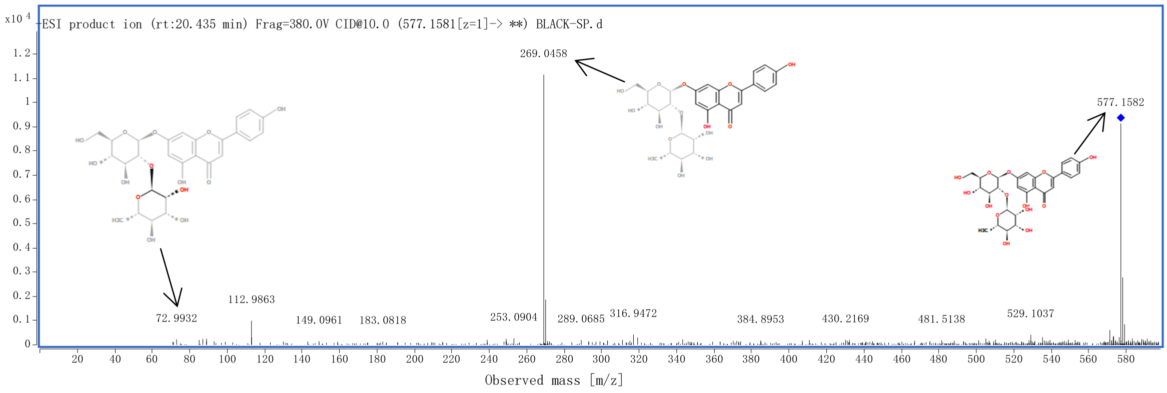

57. Galangin 3-[galactosyl-(1->4)-rhamnoside]

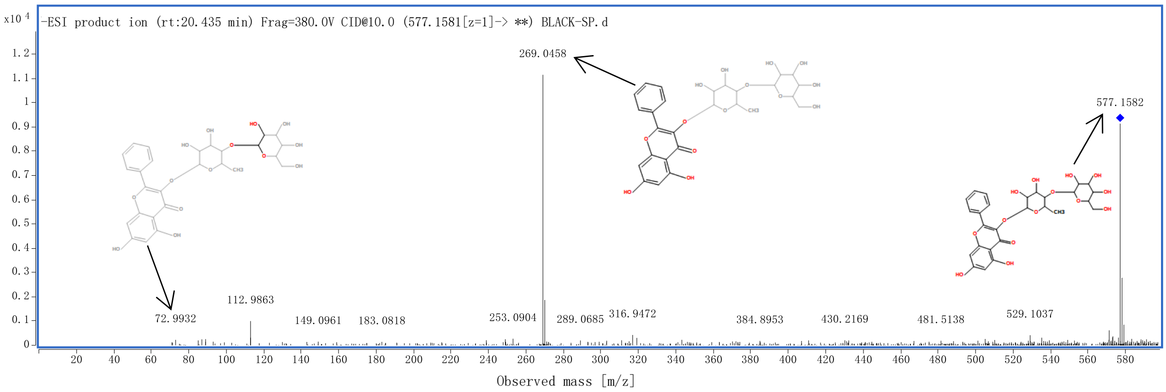

58. 6'''-O-Sinapoylsaponarin

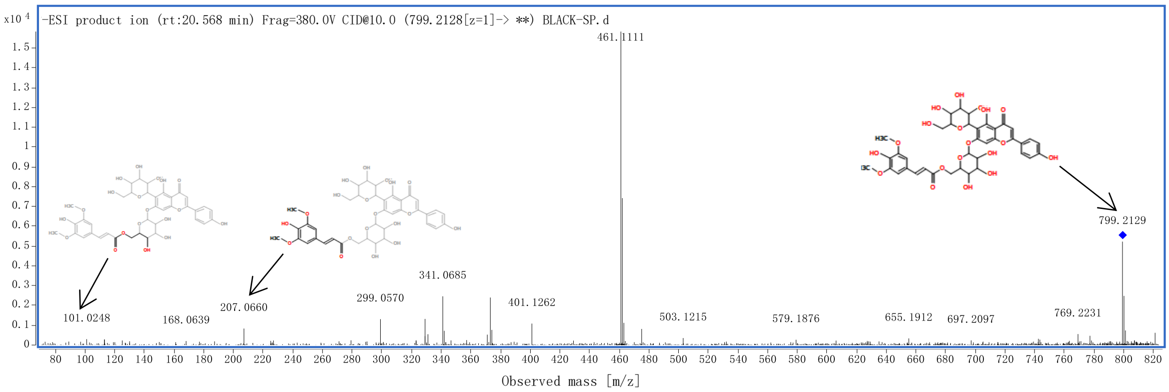

59. Isorhamnetin-3-O-neohespeidoside

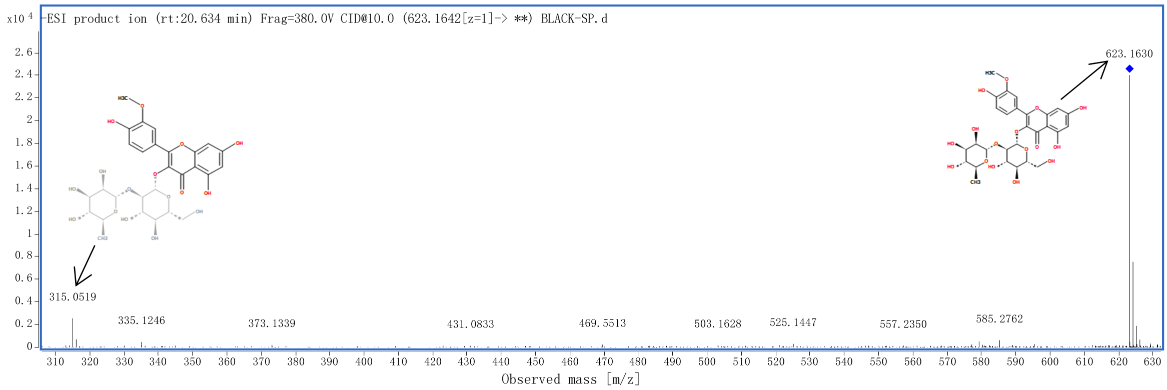

60. Isorhamnetin 3-O-[b-D-glucopyranosyl-(1->2)-a-L-rhamnopyranoside]

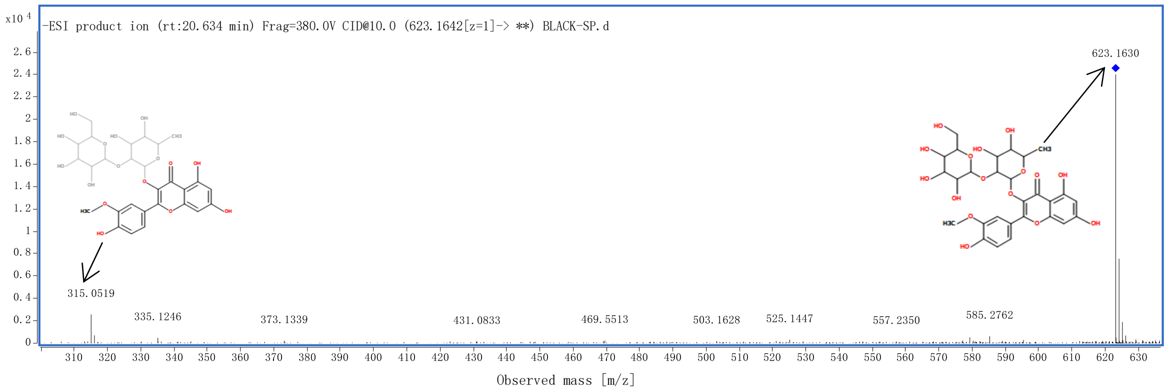

61. Spinosin

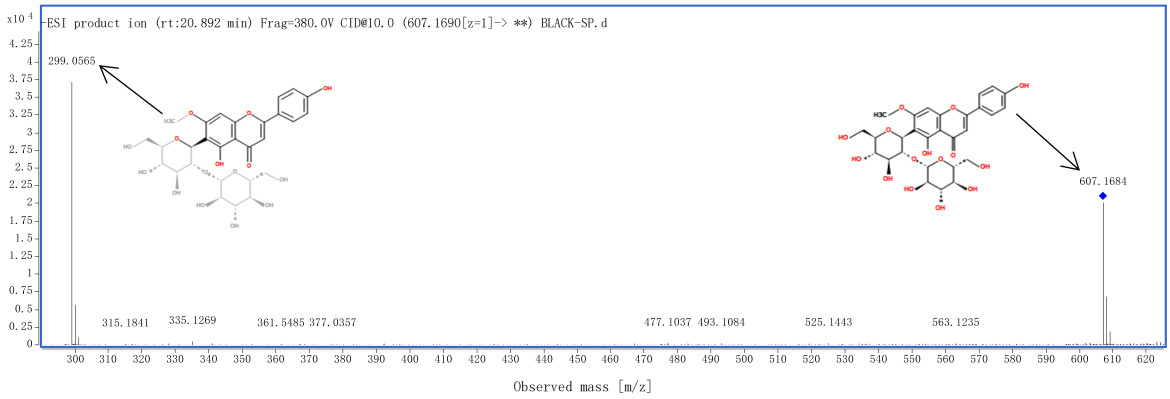

62. Diosmin

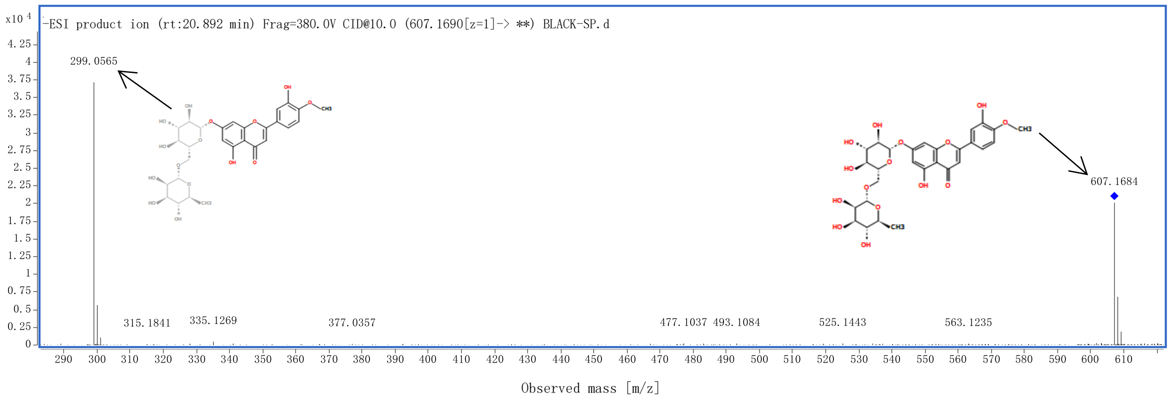

63. Quercitrin

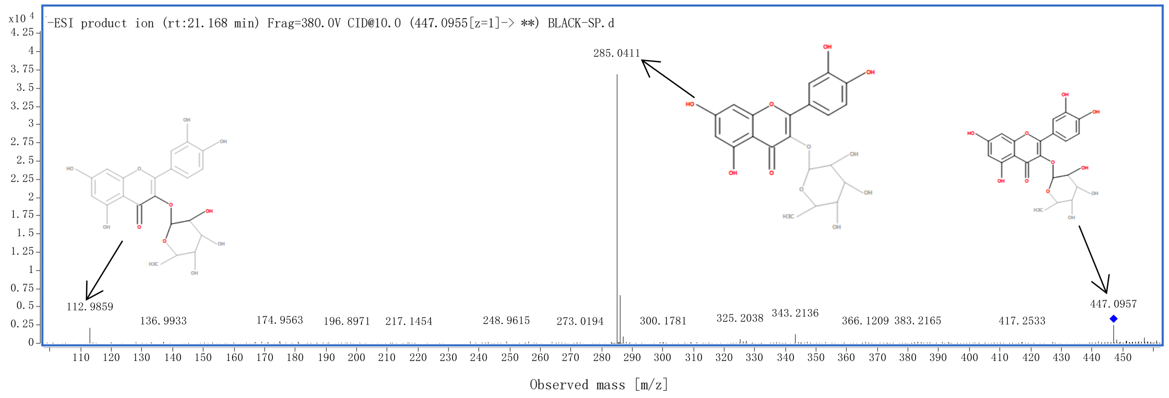

64. Scutellarin methylester

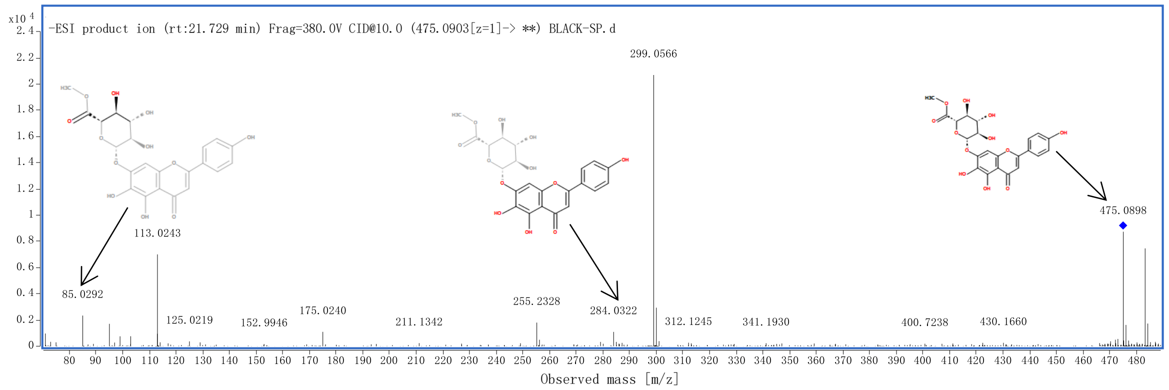

65. Diosmetin 7-O-beta-D-glucuronopyranoside

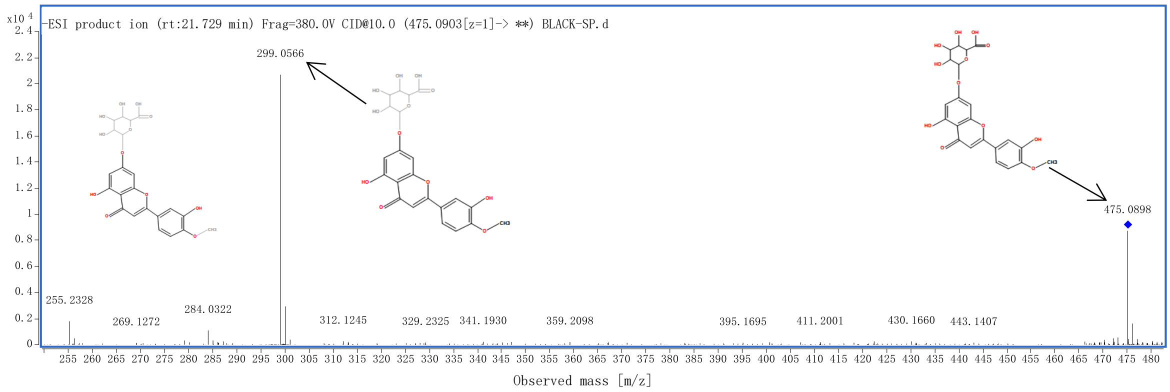

66. Tricin 7-glucuronoside

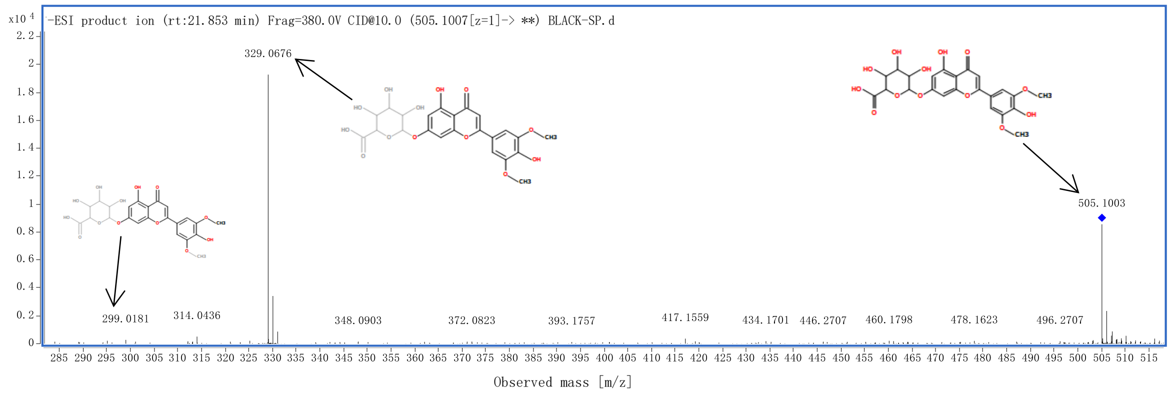

67. HESPERETIN

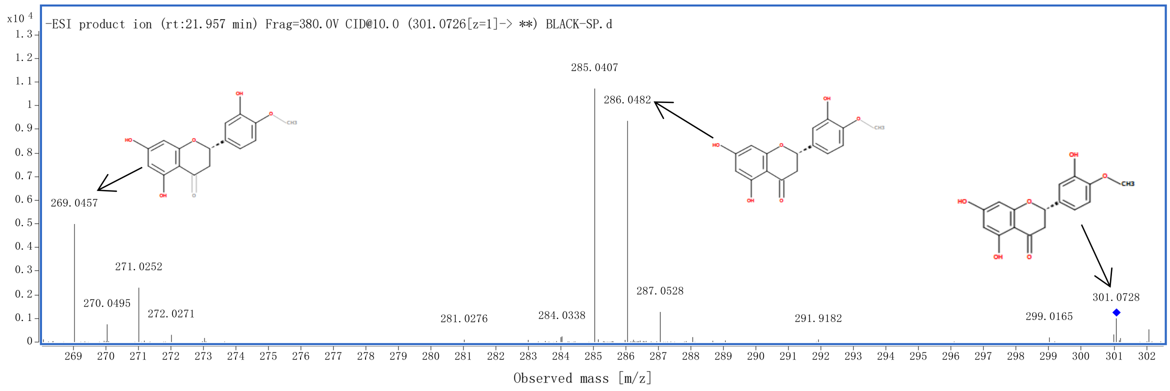

68. Tectoridin

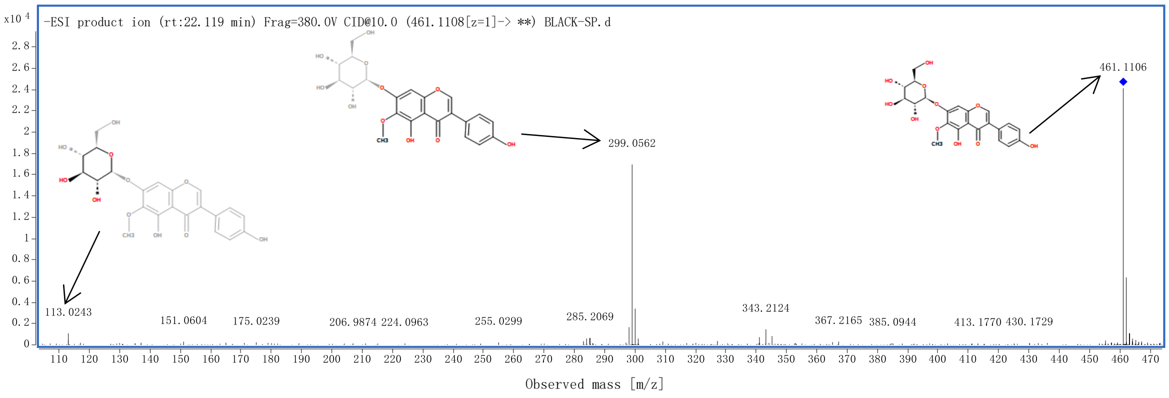

69. Kaempferide 3-galactoside

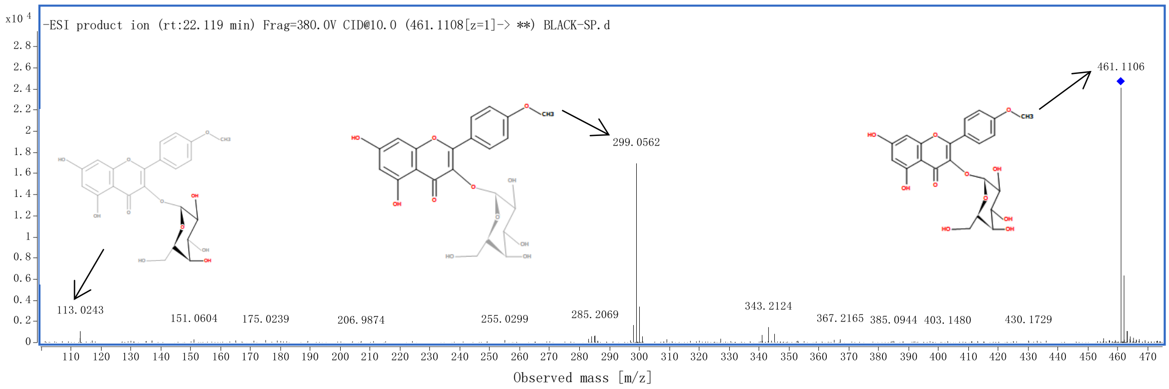

70. Isorhamnetin

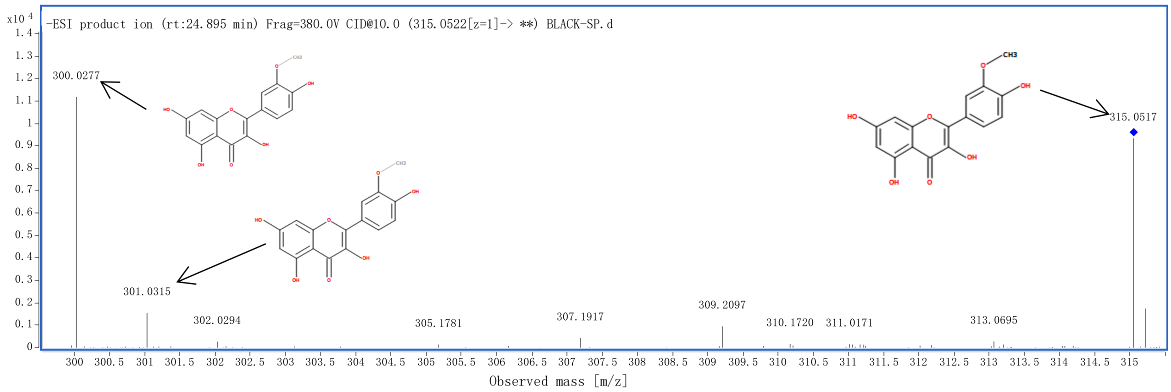

71. Petunidin

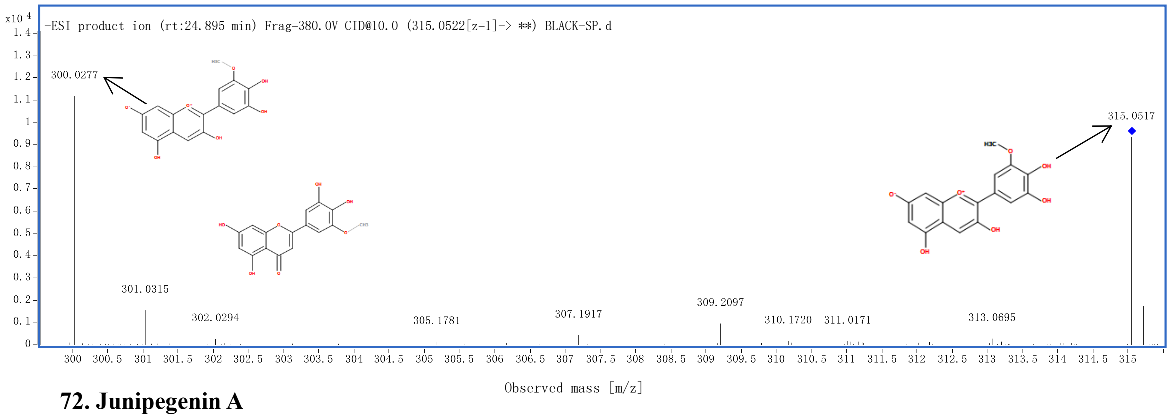

72. Junipegenin A

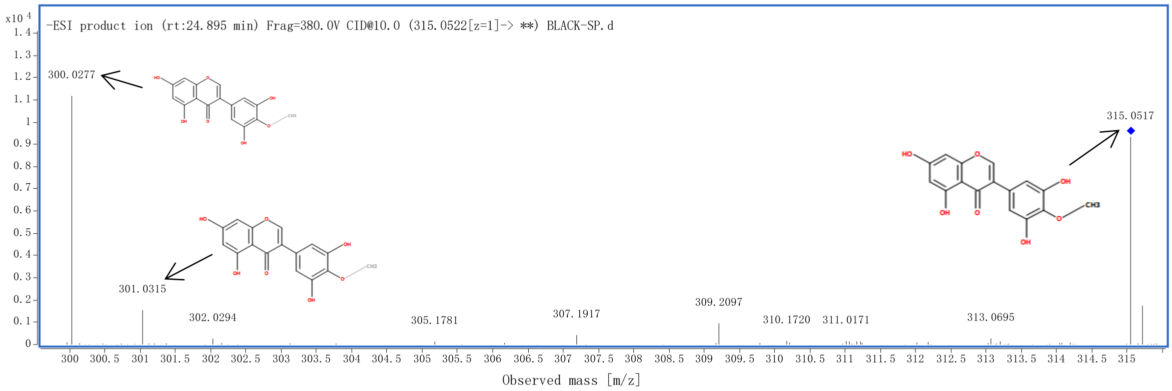

Supplementary Figure 2 The parent and daughter ion information of phenolic compounds 1-72.
